# *PoMPK3*, an MAPK Gene from Purslane (*Portulaca oleracea*), Conferred Salt Tolerance in Transgenic *Arabidopsis thaliana*

**DOI:** 10.3390/plants14223478

**Published:** 2025-11-14

**Authors:** Guoli Sun, Sunan He, Jing Dong, Tingting He, Xiaomei Zhu, Kai Wang, Zhenhua Zhang, Chong Liu, Lizhou Hong, Jincheng Xing

**Affiliations:** 1 Jiangsu Coastal Area Institute of Agricultural Sciences, Yancheng 224002, China; 20211818@jaas.ac.cn (G.S.); hsnchl@163.com (S.H.); dongjingyc@163.com (J.D.); ht142857@163.com (T.H.); xiaomeizhu301@163.com (X.Z.); wangkaimax33@163.com (K.W.); zhenhua.zhang@uwa.edu.au (Z.Z.); cellbio@163.com (C.L.); ychonglz@163.com (L.H.); 2 Key Laboratory of Saline-Alkali Soil Improvement and Utilization (Coastal Saline-Alkali Lands), Ministry of Agriculture and Rural Affairs, Nanjing 210015, China; 3 The School of Agriculture and Environment, The University of Western Australia, Perth, WA 6009, Australia

**Keywords:** *Portulaca oleracea*, PoMPK3, abscisic acid, ion homeostasis, phosphorylation

## Abstract

Mitogen-Activated Protein Kinases (MAPKs) play crucial roles in plant stress signaling, but the mechanisms of MAPK genes in *Portulaca oleracea* remain functionally uncharacterized. In this study, transcriptomic screening of *P. oleracea* under salt stress identified *PoMPK3* as a candidate gene, showing significant root-specific upregulation. Phylogenetic analysis classified it as a Group A MAPK protein, and subcellular localization confirmed its membrane association. Heterologous expression of *PoMPK3* in *Arabidopsis thaliana* significantly enhanced salt tolerance, as evidenced by improved seed germination rates, longer primary roots, increased biomass, and reduced stress symptoms. Mechanistically, *PoMPK3* expression activated ABA signaling, leading to increased ABA levels and upregulation of *AtNCED3*, *AtPYR1*, and *AtABF3*. Furthermore, it strengthened the antioxidant defense, as evidenced by elevated antioxidant enzyme activity, leading to a reduction in oxidative stress. The transgenic lines also demonstrated enhanced osmotic adjustment through osmolytes accumulation and ionic homeostasis, evidenced by tissue-specific Na^+^/K^+^ ratios (low in shoots, high in roots) resulting from the concerted upregulation of *AtSOS1*, *AtNHX1*, and *AtHKT1*. In addition, gene co-expression network analysis and molecular docking predicted phosphorylation of WRKY transcription factors, suggesting a novel mechanism for transcriptome reprogramming. Collectively, our findings not only advance the understanding of salt tolerance mechanisms in purslane but also identify *PoMPK3* as a key genetic determinant, thereby laying the foundation for its use in breeding programs aimed at enhancing salt stress resilience in crops.

## 1. Introduction

In recent years, escalating climate change has severely impacted global crop production through a combination of abiotic stresses, such as extreme temperatures, drought, and soil salinity, along with increasing biotic pressures [1]. Among these, salt stress has emerged as a global challenge, with recent assessments indicating that the proportion of irrigated agriculture impaired by salinity may significantly exceed the conventionally cited 20%, reaching over 50% in some areas [2,3]. Salt stress initially induces osmotic stress by impairing water uptake, thereby triggering physiological drought. Subsequently, excessive influx of sodium (Na^+^) and chloride (Cl^−^) ions disrupt enzymatic activity, compromises membrane integrity, and impair nutrient absorption, resulting in ion-specific toxicity and nutrient deficiency. Consequently, salt-induced metabolic dysfunction elicits an oxidative burst, resulting in the accumulation of reactive oxygen species (ROS) that inflict oxidative damage on nucleic acids [4]. Plants have evolved sophisticated regulatory networks to perceive stress signals and initiate adaptive responses, which begins with the detection of salt ions or osmotic changes by receptors on the plasma membrane, followed by second messengers and kinase cascades [5]. In response to signal activation, specific transcription factors (TFs) are mobilized, subsequently driving widespread changes in the expression of downstream target genes [6].

In plant stress signaling, the mitogen-activated protein kinase (MAPK) cascade functions as a core transducer, converting external stimuli into internal cellular responses through a conserved kinase module, including cell division, differentiation, development, hormone signaling, and programmed cell death, in addition to regulating abiotic stress tolerance [5]. Given the putative enhanced efficiency of MAPK signaling in halophytes like purslane, this may not only confer stress resilience but also contribute to its high biomass yield under adverse conditions, elevated accumulation of bioactive compounds (omega-3 fatty acids, antioxidants), and medicinal properties such as anti-inflammatory and antimicrobial activities, making it a valuable crop for saline agriculture and nutraceutical applications [7,8]. An upstream MAPKKK (MAPK kinase kinase) detects primary signals and triggers the cascade by phosphorylating MAPKK (MAPK kinase), which in turn activates MAPK through dual phosphorylation of its characteristic T-X-Y motif [9]. In *Arabidopsis thaliana*, the *AtMPK1*–*AtMKK3* cascade regulated auxin-responsive cell expansion by phosphorylating AtRBK1, which subsequently phosphorylated AtROP4 and AtROP6 to modulate cell morphogenesis [10]. In *Oryza sativa*, *OsWRKY53* functioned as a negative feedback regulator of *OsMPK3*/*OsMPK6* by interacting with these kinases and suppressing their activity, thereby modulating jasmonic acid signaling and optimizing defense resource allocation [11]. However, the functions of MPK3 and MPK6 orthologs in non-model plants, particularly in species exhibiting unique stress-resistant traits, are yet to be fully characterized. Purslane (*Portulaca oleracea*), a globally distributed halophytic vegetable and medicinal plant, thrives in saline–alkali soils, suggesting the evolution of highly efficient salt tolerance mechanisms [12]. Physiological studies have demonstrated its notable characteristics, such as efficient ion compartmentalization, active synthesis of osmotic adjustment substance, and a potent antioxidant system [7,13]. Thus, delineating the MAPK gene family in this species is crucial for understanding the unique evolutionary trajectory and functional diversity of MAPK-mediated signaling in *P. oleracea*. The MAPK gene family serves as a crucial connector between intracellular and extracellular signaling pathways and plays key roles in numerous biological processes, including the regulation of abiotic stress tolerance [9]. It has been reported that the activated MAPK cascades exhibit signal integration and amplification capabilities, enabling rapid and efficient responses to abiotic stress by orchestrating multiple downstream processes. These include ion homeostasis maintenance [14], ROS balance [15], osmotic adjustment [16], abscisic acid (ABA) signaling integration [17], and phosphorylation of TFs [18].

Therefore, we systematically assessed the expression patterns of MAPK gene family members in *P. oleracea* under salt stress, utilizing transcriptome datasets generated from three developmental stages. By comparing expression patterns across different time points of stress treatment, we identified one member exhibiting significant and sustained upregulation under salt stress. Consequently, this gene was selected and designated *PoMPK3* for deeper functional investigation. To characterize *PoMPK3* bioinformatically, we performed sequence alignment, phylogenetic reconstruction, prediction of phosphorylation sites, and subcellular localization validation. To decipher the global transcriptional regulatory network underlying salt stress response in *P. oleracea* and to elucidate the putative role of *PoMPK3*, this study integrated multiple analytical approaches, including WGCNA, enrichment analyses, and TFs–PoMPK3 interaction network reconstruction, as well as the prediction of downstream TFs. Furthermore, a comparative analysis of salt stress responses was conducted between WT and *PoMPK3* transgenic *Arabidopsis* lines to evaluate the role of *PoMPK3* in salt tolerance. Key parameters measured included seed germination rate, seedling primary root length, water loss rate, and reactive oxygen species (ROS) accumulation level. To investigate the association between *PoMPK3* and abscisic acid (ABA) signaling, we quantified endogenous ABA levels and examined the expression of key ABA-related genes. To explore the relationship between *PoMPK3* and ROS homeostasis, this study measured the activities of superoxide dismutase (SOD), catalase (CAT), and ascorbate peroxidase (APX). Additionally, to examine the regulatory function of *PoMPK3* in cell membrane permeability, we measured the accumulation level of osmotic adjustment substance. In addition, to investigate whether ion balance was affected by *PoMPK3*, we determined the ratio of Na^+^/K^+^ in *Arabidopsis* plants, as well as the expression of ion transporter genes. In summary, this study aimed to systematically characterize the function and molecular regulatory network of the core salt-tolerance gene *PoMPK3* in purslane using multi-omics and molecular biology methods. Our findings provide a framework for comparative studies of MPK3 orthologs across halophytes, with potential implications for crop improvement strategies. The observed coordination between ABA signaling and ion transporter regulation suggests *PoMPK3* may represent a convergent node in halophyte salt adaptation, though further validation of phosphorylation targets is warranted.

## 2. Materials and Methods

### 2.1. Plant Materials

In this study, seeds of purslane (salt-tolerant cultivar ‘Machixian-1’, a homozygous line) [19], preserved at the Jiangsu Coastal Area Institute of Agricultural Sciences, were cultivated in the germplasm resource nursery of the institute (33°53′ N, 120°45′ E). Upon reaching the three-leaf–one-bud stage, purslane seedlings were transferred to a controlled-environment growth chamber set to a 28 °C/20 °C (day/night) temperature cycle, 75% relative humidity, 380 µmol/mol CO_2_ concentration, and a 12 h/12 h light/dark photoperiod with a light intensity of 620 µmol·m^−2^·s^−1^. We investigated the molecular mechanisms of salt stress response in purslane by treating 20-day-old seedlings with 150 mM NaCl for 72 h, which was applied by soil irrigation with 500 mL of a 150 mM NaCl solution. This concentration was selected based on its efficacy in inducing a robust physiological response in *P. oleracea* without causing lethality during the 72 h treatment period [19] and confirmed in our pre-experiments. Control plants were irrigated with an equal volume of distilled water. Tissue samples (shoots and roots) were collected at 0 h (CK), 36 h (T1), and 72 h (T2) for transcriptome analysis. To rigorously address potential confounding effects from circadian rhythms and developmental changes [20,21], all tissue harvesting (for both salt-treated and control plants) was strictly conducted within a 2 h window during the mid-photoperiod (2:00 PM to 4:00 PM). The study contained six biological replicates (three technical replicates per sample) to ensure high data reliability.

Transformation of *PoMPK3* was performed in *A. thaliana* (common cultivar ‘Columbia-0’). The *PoMPK3* coding sequence (CDS) was cloned into the *pCAMBIA1302* expression vector under the control of the *CaMV 35S* promoter. *Arabidopsis* transformation was performed using the floral dip method [22], and homozygous T3 *A. thaliana* plants were obtained through two cycles of self-pollination, followed by hygromycin selection (50 mg·L^−1^) to ensure genetic uniformity. In addition, transgenic lines were confirmed by genomic PCR amplification and qRT-PCR analysis of *PoMPK3.* After surface sterilization (75% ethanol for 1 min, 10% sodium hypochlorite for 10 min, and rinsed five times with sterile distilled water), wild-type (WT) and transgenic seeds were germinated on MS medium (30 seeds per dish) and transferred to soil (nine plants per pot). Plants were grown in a controlled chamber at 22 °C, 60% humidity, with a 16 h light/8 h dark cycle and 54 μmol·m^−2^·s^−1^ light intensity. This standardization was critical for minimizing uncontrolled environmental variance.

### 2.2. Construction of WGCNA and Co-Expression Network

Following RNA extraction from purslane samples with the RNAiso Plus isolation Kit (Catalog No. 9108Q, Beijing Takara Biotech. Co., Ltd., Beijing, China), transcriptome library preparation and sequencing were carried out using an Illumina-compatible kit (Catalog No. 634756, Beijing Takara Biotech. Co., Ltd.) on the HiSeq 2500 platform at Gene Denovo Biotechnology Co., Ltd. (Guangzhou, China). To systematically identify co-expression modules potentially associated with *PoMPK3* from the salt stress transcriptome data of purslane, a WGCNA analysis was performed [23]. The gene expression matrix across different time points under salt stress was first normalized and filtered to remove low-expression genes. To ensure that identified genes represent robust, salt-induced responses and to minimize the inclusion of false positives arising from any residual circadian or developmental variance, stringent statistical thresholds were applied (log_2_ fold change ≥ 1.5 and adjusted *p*-value ≤ 0.01).

The WGCNA R package (version 1.72) was employed to construct a network. Key parameters included a soft-thresholding power of β = 12 (satisfy scale-free topology, R^2^ ≥ 0.85), minimum module size of 30 genes, and module merging with a cut height of 0.25. Following the construction of an adjacency matrix and its transformation into a topological overlap matrix (TOM), hierarchical clustering was applied to identify co-expression modules. Modules significantly correlated with salt stress treatment were screened by evaluating correlation coefficients and corresponding *p*-values. Genes within the selected modules were subsequently extracted for further functional annotation and network analysis. GO and KEGG enrichment analyses were conducted on the gene set comprising key modules identified by WGCNA to elucidate their potential functional roles. These were conducted using clusterProfiler package (version 4.8.1) [24], with significantly enriched terms and pathways identified based on an adjusted *p*-value threshold of ≤0.05, thereby elucidating the putative biological functions and regulatory networks of these co-expressed genes in the salt stress response. In addition, to identify TFs that may cooperate with *PoMPK3* in the salt stress response, we screened for TFs significantly co-expressed with *PoMPK3* based on WGCNA module analysis and transcriptomic co-expression data, using a Pearson correlation coefficient threshold of r > 0.8 and a *p*-value ≤ 0.05. Furthermore, by integrating known interaction databases, a *PoMPK3*–TF regulatory network was constructed using Cytoscape (version 3.10.2) [25].

### 2.3. Amplification and Bioinformatic Analysis of PoMPK3

After total RNA extraction from *P. oleracea* and *A. thaliana* using the Beyozol Total RNA Extraction Kit (Catalog No. R0011, Beyotime Biotechnology Co., Ltd., Shanghai, China), RNA quality was assessed on a NanoDrop Lite spectrophotometer (Thermo Scientific, Waltham, MA, USA). The cDNA was generated using the First Strand cDNA Synthesis Kit (Catalog No. D7180S, Beyotime Biotechnology Co., Ltd., Shanghai, China). The open reading frame (ORF) of *PoMPK3* was amplified by PCR using gene-specific primers (primer information was listed in Supplementary Table S1), and the final product was confirmed by Sanger sequencing (General Biotech Co., Ltd., Chuzhou, Anhui, China).

The CDS of *PoMPK3* from purslane and its deduced amino acid sequence were obtained through transcriptome sequencing, followed by sequence assembly, annotation, and alignment with the *P. oleracea* genome database of NCBI (BioProject No. PRJNA868526). To analyze the sequence conservation and identify potential functional sites of *PoMPK3* across distinct species, the amino acid sequence of PoMPK3 from *P. oleracea* was aligned with MPK3 homologous sequences from other plants, using ClustalW of Omega (version 1.2.4) [26]. Furthermore, to elucidate the phylogenetic position and evolutionary relationships of PoMPK3 within plants, a phylogenetic tree was constructed with MEGA 11 (version 11.0.13) [27] Neighbor-Joining algorithm to elucidate PoMPK3’s evolutionary relationship (performing 1000 bootstrap replications), while its amino acid sequence was scanned for potential serine (Ser), threonine (Thr), and tyrosine (Tyr) phosphorylation sites by the NetPhos online tool (version 3.1, https://services.healthtech.dtu.dk/services/NetPhos-3.1/ (accessed on 8 June 2025)). Similar methods have also been used to predict phosphorylation sites of potential TFs. In addition, we used AlphaFold 3 [28] to model the potential molecular interactions between *PoMPK3* and downstream TFs through protein structure prediction and docking simulations.

### 2.4. Subcellular Localization and Salt Tolerance Analysis of PoMPK3

Healthy leaves of *Nicotiana benthamiana* plants were selected for *Agrobacterium*-mediated transient gene expression analysis. The constructed *35S: GFP* empty vector (control) and *35S:PoMPK3–GFP* fusion vector were introduced into *Agrobacterium tumefaciens* strain *GV3101* (Catalog No. B528430, Sangon Biotech Co., Ltd., Shanghai, China), followed by antibiotic selection and PCR verification. Using bacterial suspensions (5 mL of overnight culture, OD_600_ = 1.0) centrifuged and resuspended in 5 mL infiltration buffer (10 mM MES, 10 mM MgCl_2_, 150 μM acetosyringone, pH 5.6) of the obtained *Agrobacterium* strains, the abaxial side of tobacco leaves was infiltrated. Following transformation, the leaf samples were subjected to fixation with 4% paraformaldehyde, permeabilization with 0.1% Triton X-100 (Catalog No. Z121066, Sangon Biotech Co., Ltd., Shanghai, China), and nuclear staining using 4′,6-diamidino-2-phenylindole (DAPI) (Catalog No. A606584, Sangon Biotech Co., Ltd., Shanghai, China). Subsequently, the samples were rinsed with phosphate-buffered saline (PBS) buffer. Subcellular localization was observed using a fluorescence microscope equipped with GFP, RFP, DAPI, and bright-field filters. Images were acquired for each channel and merged for analysis of PoMPK3–GFP protein localization in *N. benthamiana* leaf cells. This study contained three biological replicates with three technical replicates.

To validate the expression of the *PoMPK3* gene in transgenic *Arabidopsis* lines, five independent T3 homozygous overexpression lines (line #2, #9, #10, #11, and #12) were selected for analysis, with three WT plants serving as the control. The expression level of *PoMPK3* was examined using both semi-quantitative PCR and quantitative real-time PCR (qRT-PCR). In addition, the primer information of *PoMPK3* is listed in Supplementary Table S1. The seed germination assay under salt stress was performed according to an established method [29]. Following surface sterilization, seeds of WT and transgenic lines (25 per line) were sown on solid MS medium containing 0, 125, 150, or 175 mM of NaCl solutions. After a 2-day cold stratification, plates were moved to an artificial climate chamber, and germination rates were determined 4 days later.

Salt stress tolerance was further evaluated in vitro using a published method [30]. Following a 12-day growth period on solid MS medium with or without 150 mM NaCl, root lengths of WT and transgenic seeds (five per line) were quantified and statistically evaluated. Additionally, salt stress resistance in *Arabidopsis* seedlings was assessed as previously described [31]. One-week-old *Arabidopsis* seedlings (4 per pot) were transplanted into pots filled with an autoclaved soil mixture and grown in a growth chamber. After a two-week acclimation period, salt treatment was initiated by irrigating with 150 mM NaCl every two days for two weeks. This volume was sufficient to achieve thorough soil saturation and ensure drainage from the bottom of the pot. The plants were grown in plastic pots, each containing a total of 3.0 kg of a soil mixture (soil:vermiculite:perlite = 3:1:1, *v*/*v*). Phenotypic traits (including water loss rate, relative electrical conductivity, and total chlorophyll content) [32,33] were systematically evaluated. This study contained three biological replicates with three technical replicates.

### 2.5. Determination of Physiological Indices and Gene Expression in Arabidopsis Salt Stress Resistance

The determination of chlorophyll content and relative electrical conductivity (REC) in *Arabidopsis* leaves followed a previous report [34]. For chlorophyll extraction, leaf powder was incubated with 80% acetone in the dark at 4 °C. After centrifugation (4 °C, 12,000× *g*, 15 min), the supernatant’s absorbance was measured at 663 and 645 nm for calculating chlorophyll a, b, and total chlorophyll concentrations. REC was measured using a DDS-307 conductivity meter (Shanghai INESA, Shanghai, China) after vacuum-infiltrating leaf discs in deionized water for 30 min to remove initial electrolytes. After incubation, the initial electrical conductivity (C_1_) of the solution was measured, followed by boiling the samples for 20 min and measuring the final conductivity (C_2_). REC was calculated as (C_1_/C_2_) × 100%.

Endogenous ABA levels in both WT and *PoMPK3* transgenic *Arabidopsis* lines were quantified using the HPLC-MS method [35]. Oxidative stress markers (O_2_^−^, H_2_O_2_, MDA), and antioxidant enzyme activities (SOD, CAT, APX) in both WT and *PoMPK3* transgenic *Arabidopsis* lines were determined using the methods described before [36]. Furthermore, osmolyte (proline, glycine betaine, soluble sugars and proteins) levels in *Arabidopsis* were determined according to the method [37,38].

Determination of sodium ion (Na^+^) and potassium ion (K^+^) levels in *A. thaliana* lines was performed using the method with minor modifications [39]. To prepare for elemental analysis, the harvested leaves and roots were washed with distilled water and dried to a constant weight in the 80 °C oven over a 72 h period. 0.05 g portions of the homogenized dry powder were weighed for acid digestion. The digestion process utilized 5 mL of concentrated HNO_3_ (69%, *v*/*v*) and comprised an overnight pre-digestion at room temperature followed by a controlled microwave-assisted digestion at 200 °C to achieve complete dissolution. After thorough acid removal by evaporation, the samples were made up to 50 mL with a 2% HNO_3_ solution. Concentrations of sodium (^23^Na) and potassium (^39^K) were determined by ICP-MS (PerkinElmer, Waltham, MA, USA), applying internal standardization with scandium (Sc) and yttrium (Y) to mitigate matrix-related inaccuracies. In addition, all physiological data are presented on a fresh weight (FW) basis.

The expression levels of *AtNCED3* (NCBI accession No. NM_112304.3, encoding 9-cis-epoxycarotenoid dioxygenase 3) [40], *AtPYR1* (NCBI accession No. NM_117896.3, encoding pyrabactin resistance 1) [41], and *AtABF3* (NCBI accession No. NM_001342246.1, encoding ABRE-binding factor 3) [42] were assayed to precisely delineate the functional node of *PoMPK3* within the ABA signaling pathway. Additionally, the expression of *AtSOS1* (NCBI accession No. AF256224.1, encoding salt overly sensitive 1) [43], *AtHKT1* (NCBI accession No. NM_117099.6, encoding high-affinity K^+^ transporter 1) [44], and *AtNHX1* (NCBI accession No. NM_122597.3, encoding Na^+^/H^+^ exchanger 1) [45] was measured to the role of *PoMPK3* in regulating ion balance. In addition, *AtUBQ10* (NCBI accession No. NM_001084884.5, encoding Ubiquitin 10) and *AtPP2A* (NCBI accession No. NM_001331905.1, encoding protein phosphatase 2A) were used as reference genes of *Arabidopsis* [46], *PoACT7* (Phytozome accession No. FUN_033957, encoding actin 7) and *PoTUB* (Phytozome accession No. FUN_003921, encoding tubulin) were used as reference genes of *P. oleracea*, and the geometric mean of their expression levels was used for relative quantification of the target genes. The primer information (including primer nucleotide sequence, amplified fragment size, and Tm) for these target and reference genes is listed in Supplementary Table S1. Additionally, these studies contained three biological replicates with three technical replicates.

### 2.6. Statistical Analysis

Differences in gene expression and physiological indices among developmental stages of *P. oleracea* and *A. thaliana* lines were evaluated using Student’s *t*-test and one-way ANOVA. Data visualization was conducted with GraphPad Prism (Version 8.0, https://www.graphpad.com/ (accessed on 7 June 2025).). For multiple comparisons, lowercase letters (a, b, c) were assigned to indicate significant differences at *p* ≤ 0.05. To minimize cumulative error, three or more independent biological replicates were included per treatment, with each consisting of three technical replicates. In addition, a flowchart describing methodology of this study is shown in Supplementary Figure S1.

## 3. Results

### 3.1. Screening and Bioinformatics Analysis of PoMPK3

**Figure 1:**
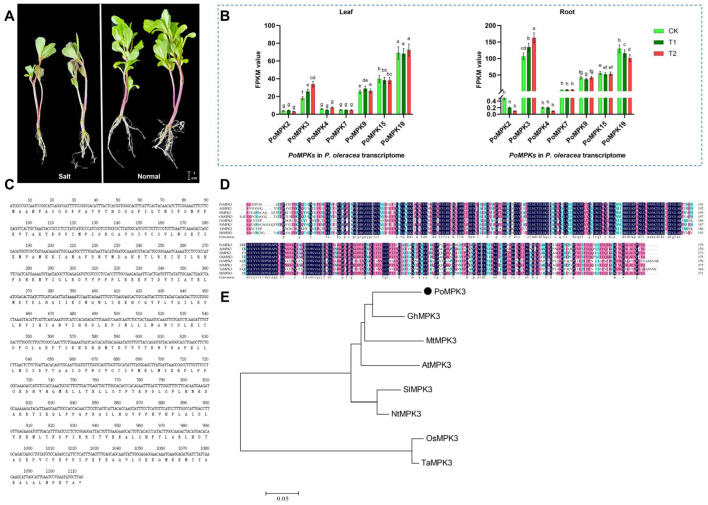
Genome-wide identification of the MPK gene family and functional characterization of *PoMPK3* in *P. oleracea*. (**A**) Phenotypic comparison of mature *P. oleracea* plants grown under control (0 mM NaCl) and salt stress (200 mM NaCl for 7 days). Scale bar = 1 cm. (**B**) Heatmap of expression profiles (FPKM values) for seven identified *PoMPKs* in leaf and root tissues of *P. oleracea* under salt stress at three time points (CK, T1, T2). The significance was indicated using the a, b, c, d, e, f, g, h letter, with a *p* value ≤ 0.05 considered significant. (**C**) Basic characteristics of the *PoMPK3* CDS and its encoded protein. (**D**) Amino acid sequence alignment of PoMPK3 protein with homologs from six species, including AtMPK3 (No. NP_190150.1) of *Arabidopsis thaliana*, SlMPK3 (NP_001234360.1) of *Solanum lycopersicum*, GhMPK3 (NP_001314498.1) of *Gossypium hirsutum*, OsMPK3 (NP_001396248.1) of *Oryza sativa*, NtMPK3 (XP_070052446.1) of *Nicotiana tomentosiformis*, TaMPK3 (XP_044418724.1) of *Triticum aestivum*, and MtMPK3 (XP_024637805.2) of *Medicago truncatula.* These sequences were aligned using ClustalW of MEGA 11.0, and conserved regions were shaded. (**E**) *NJ* phylogenetic tree (1000 bootstrap replicates) constructed based on the sequences of PoMPK3 protein clustering with its homologs. The scale bar represented 0.05 amino acid substitutions per site.

Based on the transcriptome sequencing database (NCBI accession No. PRJNA1290847) of *P. oleracea* under salt stress at three time points (CK, T1, T2) (Figure 1A), we identified seven genes belonging to the PoMPKs family (Supplementary Figure S2). These genes were designated as *PoMPK2*, *PoMPK3*, *PoMPK4*, *PoMPK7*, *PoMPK9*, *PoMPK15*, and *PoMPK19* according to NCBI BLAST (version 2.17.0) results. Analysis of the transcriptomic data for the MPK gene family in *P. oleracea* (Figure 1B) revealed significant differences in expression abundance (FPKM values) among family members under salt stress. Notably, *PoMPK3* exhibited markedly higher expression levels, significantly exceeding both the median and mean expression values of other *PoMPKs*. Specifically, *PoMPK3* expression exhibited distinct tissue specificity, with significantly higher FPKM values in root tissues compared to leaf tissues, suggesting its functional relevance is primarily associated with root-related physiological processes. Meanwhile, under salt stress, *PoMPK3* expression in roots showed a consistent upregulation trend relative to CK, indicating its potential involvement in salt stress response in the root system of *P. oleracea*. Therefore, *PoMPK3* represented a core member of the MPK gene family, and its expression profile suggested that it functioned as a key candidate gene, warranting further functional validation.

Bioinformatic analysis revealed that the CDS of *PoMPK3* comprised 1110 nucleotides, encoding a protein of 370 amino acids with a theoretical isoelectric point (pI) of 5.78, indicating that PoMPK3 was an acidic protein (Figure 1C). To characterize the sequence features of *PoMPK3*, we conducted multiple sequence alignment of its CDS with *MAPK3* homologs from six closely related species (*Arabidopsis thaliana*, *Solanum lycopersicum*, *Gossypium hirsutum*, *Oryza sativa*, *Nicotiana tomentosiformis*, *Triticum aestivum*, and *Medicago truncatula*). Clustal analysis (Figure 1D) demonstrated a striking evolutionary conservation, particularly within the critical activation loop (T-E-Y motif) of the kinase domain. Among these, the amino acid sequence of PoMPK3 showed the highest similarity with GhMPK3 (83.38%). Meanwhile, to clarify the evolutionary position of the *PoMPK3* gene from *P. oleracea* within the MPK family, we constructed a phylogenetic tree using its amino acid sequence and MPK3 homologs from representative plant species, including AtMPK3 (NCBI accession No. NP_190150.1) of *Arabidopsis thaliana*, SlMPK3 (NP_001234360.1) of *Solanum lycopersicum*, GhMPK3 (NP_001314498.1) of *Gossypium hirsutum*, OsMPK3 (NP_001396248.1) of *Oryza sativa*, NtMPK3 (XP_070052446.1) of *Nicotiana tomentosiformis*, TaMPK3 (XP_044418724.1) of *Triticum aestivum*, and MtMPK3 (XP_024637805.2) of *Medicago truncatula*. It was found that all known MAPK3 homologs clustered into a distinct, well-supported evolutionary branch, and PoMPK3 was accurately grouped within this clade and exhibited the closest relationship with GhMAPK3 (Figure 1E). These results confirmed at the phylogenetic level that the *PoMPK3* gene we amplified was an ortholog of MAK3 in *P. oleracea*.

### 3.2. Functional Exploration of PoMPK3 in Response to Salt Stress in Arabidopsis

**Figure 2:**
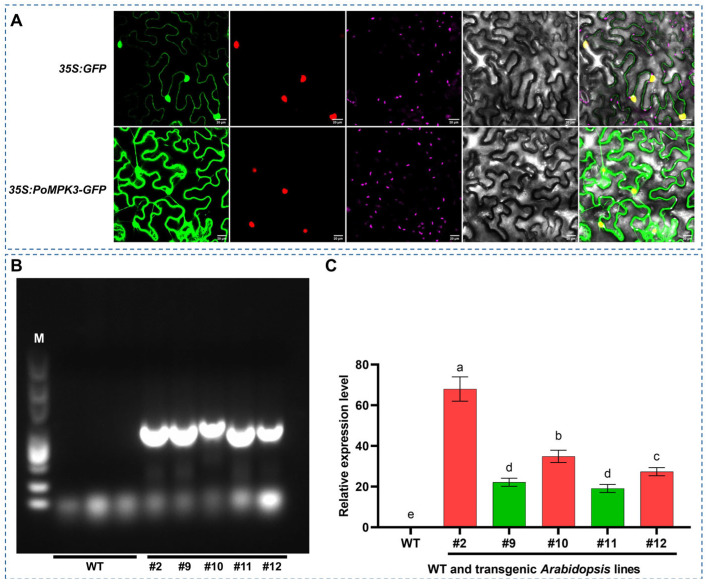
Subcellular localization of PoMPK3 and molecular identification of transgenic *Arabidopsis* lines. (**A**) Subcellular localization analysis of *35S:PoMPK3–GFP* and *35S:GFP* (control) in *N. benthamiana* epidermal cells. Confocal microscopy images were captured at 48 h post-agroinfiltration (scale bar = 20 μm). Images were acquired using a fluorescence microscope with 488 nm (GFP) and 405 nm (DAPI) laser lines. (**B**) Genomic PCR verification of transgenic *A. thaliana* lines (line #2, #9, #10, #11, and #12), and M indicated DNA ladder marker. (**C**) qRT-PCR analysis of *PoMPK3* relative expression levels in leaves of WT and transgenic *A. thaliana* lines (line #2, #9, #10, #11, and #12). Data represents SD (*n* = 3 biological replicates), with each experiment consisting of technical triplicates. The significance was indicated using the a, b, c, d, e letter, with a *p* value ≤ 0.05 considered significant.

To investigate the location and distribution of the PoMPK3 protein within plant cells, subcellular localization analysis was conducted by expressing a *35S:PoMPK3–GFP* fusion construct. The DAPI staining (purple) clearly delineated the nuclear compartments. The GFP fluorescence signal (green) exhibited a distinct and continuous outlining pattern, precisely defining the plasma membrane and intracellular membrane structures. Critically, in the merged channel image, the green fluorescence from PoMPK3–GFP showed a clear separation from the purple nuclear signal, with no observable overlap. This result provides direct visual evidence that PoMPK3 is specifically targeted to the membrane system, effectively excluding the possibility of nuclear localization. In contrast, the control experiment with *35S: GFP* alone showed diffuse fluorescence throughout the cytoplasm and nucleus (Figure 2A). These findings conclusively demonstrated that PoMPK3 undergoes specific membrane trafficking and possesses an intrinsic membrane-targeting signal, which indicated its unique subcellular distribution pattern.

To functionally characterize the role of *PoMPK3* in salt tolerance, stable transgenic *A. thaliana* lines overexpressing *PoMPK3* were generated through *A. tumefaciens*-mediated transformation. Homozygous T3 generation lines were obtained through self-pollination and antibiotic selection. As shown in Figure 2B, genomic PCR analysis using primers specific to *PoMPK3* confirmed the successful integration of the transgene into the genome of multiple independent lines (line #2, #9, #10, #11, #12), as evidenced by the presence of the expected amplicon, which was absent in WT. In addition, the expression level of *PoMPK3* was quantitatively assessed by qRT-PCR. As shown in Figure 2C, substantial accumulation of *PoMPK3* was detected in the leaves of all transgenic lines. Notably, transgenic lines #2, #10, and #12 exhibited the most pronounced overexpression levels, establishing them as ideal candidates for subsequent phenotypic screening.

**Figure 3:**
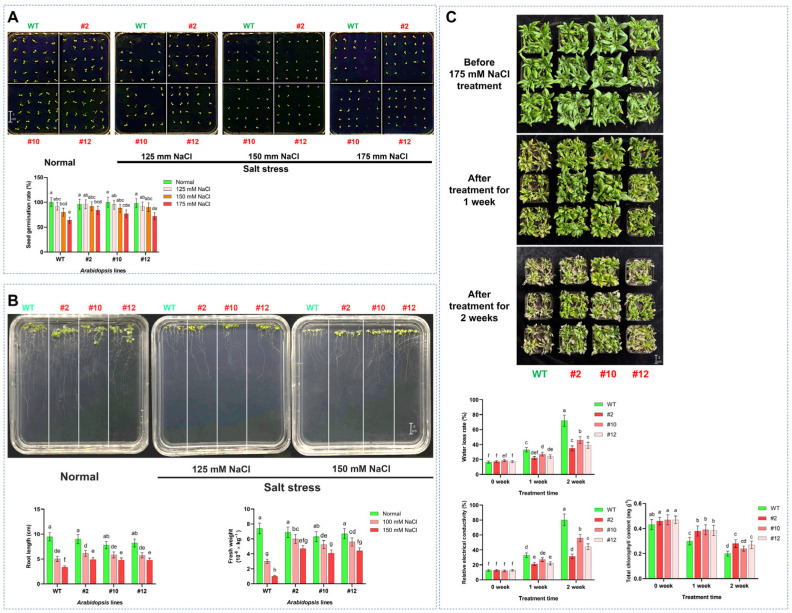
Comprehensive analysis of salt tolerance conferred by *PoMPK3* overexpression in *A. thaliana*. (**A**) Seed germination assay under salt stress. Seeds of WT and transgenic lines (T3 homozygous lines #2, #10, #12) were surface-sterilized and germinated on MS medium containing 0, 125, 150, or 175 mM NaCl (*n* = 25 seeds per plate, 3 replicates). Germination rates were recorded after 4 days. (**B**) Phenotypic and physiological profiling of *Arabidopsis* seedlings. Growth phenotypes of WT and transgenic seedlings under normal and salt-stress conditions were shown. Primary root length (cm) and fresh weight (10^−6^ kg^−1^) were measured after 7-day exposure to 0, 125, or 150 mM NaCl (*n* = 5 seedlings per line). (**C**) Physiological responses of mature *Arabidopsis* plants under salt stress. The changes in key physiological indices (water loss rate, relative electrical conductivity, and total chlorophyll content) were assayed (*n* = 3 biological replicates, each with 3 technical replicates). The significance was indicated using the a, b, c, d, e, f, g, h letter, with a *p* value ≤ 0.05 considered significant.

The germination assay under salt stress revealed a concentration-dependent suppression of germination across all *Arabidopsis* genotypes after 4 days. While the germination rates of WT and transgenic lines (line #2, #10, and #12) decreased with increasing NaCl concentrations (125 mM, 150 mM, and 175 mM). At the same time, the transgenic lines, particularly #2, maintained significantly higher germination rates compared to WT under salt stress (175 mM NaCl). It was found that transgenic line #2 exhibited significantly higher seed germination rates compared to WT under NaCl stress, with increases of 4.35% at 125 mM, 15.33% at 150 mM, and 31.25% at 175 mM NaCl concentration (Figure 3A). To further characterize the salt tolerance conferred by *PoMPK3* in *A. thaliana*, phenotypic and physiological analyses were conducted on WT and transgenic *Arabidopsis* seedlings grown under normal and salt-stressed conditions. While no significant growth difference was observed on standard MS medium without salt treatment (normal condition), distinct phenotypic variations emerged under 125 mM and 150 mM NaCl solution treatments. It was observed that the transgenic *Arabidopsis* plants (line #2, #10, and #12) overexpressing *PoMPK3* significantly enhanced growth vigor compared to WT, reflected by longer primary roots and increased fresh weight. Quantitative assessment showed that the root lengths of transgenic lines #2, #10, and #12 were significantly greater than those of WT, with line #2 displaying increases of 21.63% under 125 mM NaCl and 46.11% under 150 mM NaCl. A corresponding significant increase in fresh weight was also observed in the transgenic lines under salt stress (Figure 3B). In addition, to further validate the regulatory function of *PoMPK3* in plant salt stress tolerance, in vivo assays were conducted comparing WT and *PoMPK3* transgenic *Arabidopsis* lines. No significant morphological differences were observed between WT and *PoMPK3* transgenic lines before salt stress treatment. After one week of exposure to 175 mM NaCl, WT plants began to show clear stress symptoms, including reduced rosette size and initial signs of chlorosis. In contrast, transgenic lines maintained healthier growth, with line #2 exhibiting the mildest symptoms. Following two weeks of salt treatment, the phenotypic differences became more pronounced. WT plants displayed severe growth inhibition, extensive chlorosis, and widespread leaf wilting. In contrast, the transgenic lines continued to demonstrate superior salt tolerance, characterized by significantly larger rosettes, higher biomass accumulation, and retention of green, turgid leaves. Throughout the stress period, line #2 showed the best growth performance among all transgenic lines (Figure 3C).

Furthermore, we also measured several stress-related physiological indices (including water loss rate, relative electrical conductivity, and total chlorophyll content) in both WT and *PoMPK3* transgenic lines at different stages of treatment. The data showed that after 1 week of salt stress, the water loss rate of WT plants increased to 33.33%, while transgenic lines #2, #10, and #12 exhibited rates of 22.15%, 27.31%, and 24.46%, respectively. Two weeks later, the water loss rate of WT reached 72.66%. In contrast, transgenic lines #2, #10, and #12 showed significantly lower rates of 35.17%, 46.56%, and 39.23%, respectively. We found a similar pattern of change in relative electrical conductivity. After 2 weeks of salt stress, the relative electrical conductivity of WT reaches 79.98%, and transgenic lines #2, #10, and #12 maintain significantly lower levels of 31.34%, 55.86%, and 44.19%, respectively. In addition, it was found that after 2 weeks of salt stress, the chlorophyll content of WT declined to 0.21 mg g^−1^, while *PoMPK3* transgenic lines #2, #10, and #12 retained significantly higher levels of 0.28 mg g^−1^, 0.24 mg g^−1^, and 0.27 mg g^−1^, respectively (Figure 3C).

### 3.3. Regulatory Mechanism Underlying PoMPK3-Mediated Salt Tolerance in Arabidopsis Plants

**Figure 4:**
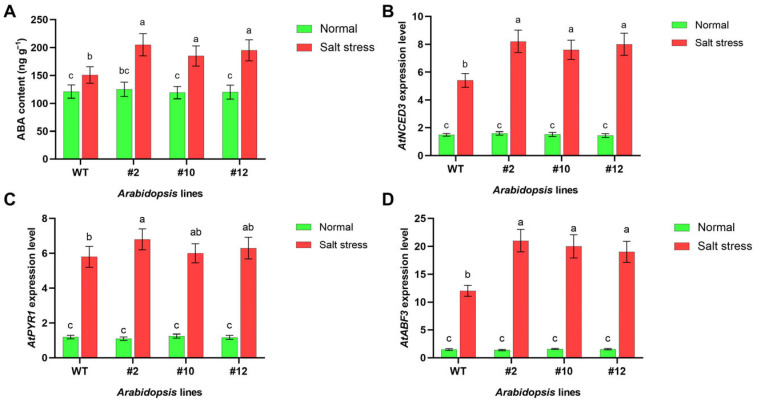
*PoMPK3*-mediated enhancement of ABA signaling contributes to salt tolerance. (**A**) Endogenous ABA levels in leaves of WT and transgenic lines (line #2, #10, and #12) under normal and salt (175 mM NaCl solution) conditions. Relative expression levels of key ABA metabolism genes in leaves of WT and transgenic lines under normal and salt-stress conditions, including (**B**) *AtNCED3* (ABA biosynthesis gene), (**C**) *AtPYR1* (ABA receptor gene), and (**D**) *AtABF3* (ABRE-binding factor). *AtUBQ10* and *AtPP2A* served as reference genes. Data represents SD (*n* = 3 biological replicates), with each experiment consisting of technical triplicates. The significance was indicated using the a, b, c letter, with a *p* value ≤ 0.05 considered significant.

Following a 24 h exposure to sterile water (control) or 175 mM NaCl (salt stress) in pot culture, leaves and roots were collected from two-week-old WT and *PoMPK3* transgenic *Arabidopsis* seedlings for analysis. To explore whether *PoMPK3* regulated salt tolerance of *Arabidopsis* plants by modulating ABA metabolism, we examined endogenous ABA content and ABA-related gene expression levels. As shown in Figure 4A, salt stress treatment triggered ABA accumulation in both WT and *PoMPK3* overexpressing *Arabidopsis* seedlings, with transgenic lines exhibiting a more pronounced response, and the endogenous ABA content was significantly higher in the transgenic lines compared to WT under salt stress. Notably, line #2 showed the most significant increase, with ABA levels reaching 1.36 times that of WT. Meanwhile, at the transcriptional level, salt stress significantly increased the expression of key ABA pathway genes, including the ABA biosynthesis gene *AtNCED3* (Figure 4B), the ABA receptor gene *AtPYR1* (Figure 4C), and the downstream ABRE-binding factor *AtABF3* (Figure 4D). Under normal conditions, transcript levels of these genes in lines #2, #10, and #12 were similar to those in WT. However, salt stress significantly induced their expression in all transgenic lines, with *AtPYR1* showing the most prominent upregulation in line #2, significantly exceeding that in lines #10 and #12. These results indicated that *PoMPK3* expression effectively activated the ABA signaling pathway in *Arabidopsis* under salt stress, as demonstrated by increased endogenous ABA levels and upregulation of key pathway genes.

**Figure 5:**
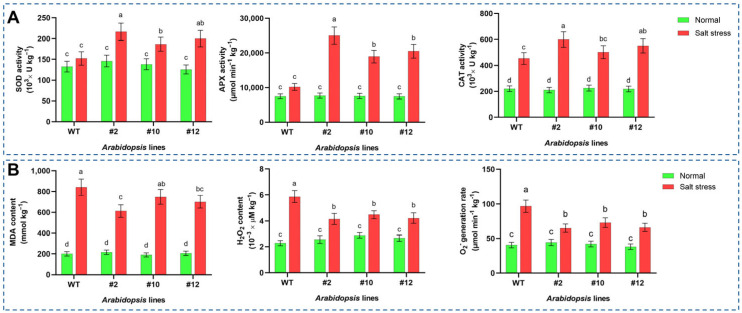
Analysis of antioxidant enzyme activities and oxidative damage indices in *PoMPK3* transgenic *Arabidopsis* plants under salt stress. (**A**) Activities of key antioxidant enzymes (SOD, APX, CAT) in leaves of WT and *PoMPK3* transgenic *Arabidopsis* plants (line #2, #10, #12) after 7-day exposure to 175 mM NaCl. (**B**) Levels of oxidative damage indices (MDA, H_2_O_2_, O_2_^−^) in leaves of WT and *PoMPK3* transgenic *Arabidopsis* plants (line #2, #10, #12) after 7-day exposure to 175 mM NaCl. Data represents SD (*n* = 3 biological replicates), with each experiment consisting of technical triplicates. The significance was indicated using the a, b, c, d letter, with a *p* value ≤ 0.05 considered significant.

To investigate whether *PoMPK3* enhanced salt tolerance by modulating ABA-mediated ROS homeostasis, we quantified the accumulation levels of MDA, H_2_O_2_, and O_2_^−^, along with the activities of SOD, CAT, and APX in *Arabidopsis*. As shown in Figure 5A, under salt stress, SOD activity in WT increased to approximately 146.34 × 10^3^ U kg^−1^. The transgenic lines exhibited significantly higher activities, with #2, #10, and #12 reaching 216.53 × 10^3^, 180.16 × 10^3^, and 194.21 × 10^3^ U kg^−1^, respectively. This represents an increase of 47.96%, 23.39%, and 32.88% compared to WT under stress. In addition, salt stress induced APX activity in WT to 10,189.253 μmol min^−1^ kg^−1^. The transgenic lines showed markedly higher induction, with activities of 25,032.71, 18,938.21, and 20,485.64 μmol min^−1^ kg^−1^ for lines #2, #10, and #12, respectively. Meanwhile, under stress, CAT activity in WT remained largely unchanged at 452.88 × 10^3^ U kg^−1^. In contrast, the transgenic lines displayed significant induction, with activities rising to 599.76, 501.84, and 550.81 × 10^3^ U kg^−1^ for lines #2, #10, and #12, representing 32.74%, 10.84%, and 21.68% increases over WT. In addition, all *PoMPK3* transgenic lines accumulated significantly lower levels of oxidative damage indices (MDA, H_2_O_2_, and O_2_^−^) compared to WT. Line #2 exhibited the most significant reduction in all three indices, consistent with its highest antioxidant enzyme activities (Figure 5B).

**Figure 6:**
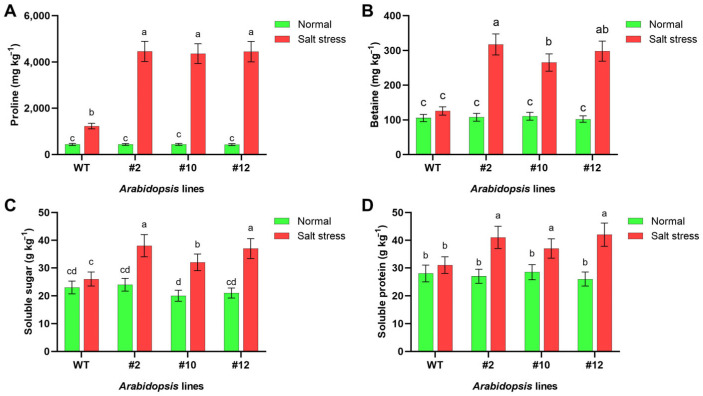
Effects of *PoMPK3* expression on osmotic regulation in *A. thaliana* plants under salt stress. (**A**) Proline content, (**B**) betaine content, (**C**) soluble sugar content, and (**D**) soluble protein content in leaves of WT and transgenic lines (#2, #10, #12) under control (0 mM NaCl) and salt stress (175 mM NaCl) for 7 days. Data represents SD (*n* = 3 biological replicates), with each experiment consisting of technical triplicates. The significance was indicated using the a, b, c, d letter, with a *p* value ≤ 0.05 considered significant.

The levels of proline, betaine, soluble sugar and protein were quantified in both WT and transgenic *Arabidopsis* to assess the contribution of *PoMPK3* to osmotic adjustment. It was found that under salt stress, proline content increased significantly in all *Arabidopsis* lines. WT proline levels rose from 437.504 mg·kg^−1^ under normal conditions to 1231.23 mg·kg^−1^ under stress. However, transgenic lines showed more pronounced accumulation, with line #2 reaching 4458.48 mg·kg^−1^, which was 3.63 times that of WT, and 1.13 and 1.06 times that of lines #10 and #12, respectively (Figure 6A). Similar to proline, betaine content in *Arabidopsis* increased significantly under salt stress. WT levels rose from 105.35 mg·kg^−1^ to 125.16 mg·kg^−1^, while transgenic lines showed substantially higher accumulation. Line #2 reached mg·kg^−1^, representing 2.54 times that of WT, and 1.19 and 1.06 times that of lines #10 and #12, respectively (Figure 6B). In addition, salt stress induced more significant accumulation of soluble sugar and soluble protein in *PoMPK3* transgenic *Arabidopsis* lines compared to WT. Specifically, under salt stress, line #2 exhibited 61.533% higher soluble sugar content than WT (Figure 6C), while line #12 showed a 35.48% increase in soluble protein content relative to WT (Figure 6D).

### 3.4. PoMPK3 Modulated Ion Homeostasis to Confer Salt Tolerance in Arabidopsis

**Figure 7:**
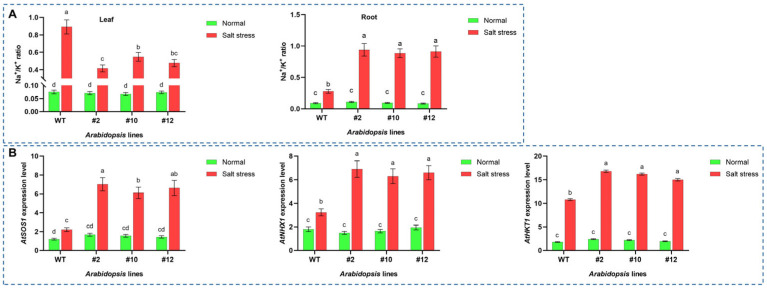
PoMPK3 enhanced salt tolerance by regulating key ion transporters and maintaining Na^+^/K^+^ homeostasis. (**A**) Quantification of Na^+^/K^+^ ratios in leaves and roots of WT and *PoMPK3* transgenic lines (line #2, #10, #12) under control (0 mM NaCl) and salt stress (175 mM NaCl) for 7 days. (**B**) Relative transcript abundance of key ion transporter genes (*AtSOS1*, *AtNHX1*, *AtHKT1*) in leaves of WT and transgenic lines under salt stress (175 mM NaCl treatment for 24 h). qRT-PCR was performed using SYBR Green Master Mix (Takara), with *AtUBQ10* and *AtPP2A* as the reference genes. Data represents SD (*n* = 3 biological replicates), with each experiment consisting of technical triplicates. The significance was indicated using the a, b, c, d letter, with a *p* value ≤ 0.05 considered significant.

To elucidate the role of *PoMPK3* in regulating ion homeostasis, we measured Na^+^ and K^+^ concentrations and analyzed the expression of key ion transporter genes in leaf and root tissues of WT and *PoMPK3* transgenic *Arabidopsis* lines (line #2, #10, #12) under salt stress. Ion content analysis revealed that *PoMPK3* expression in *Arabidopsis* induced organ-specific ion distribution patterns under salt stress. In *Arabidopsis* leaf tissues, transgenic lines, particularly line #2, exhibited a ‘low Na^+^/high K^+^’ pattern, with significantly lower Na^+^/K^+^ ratios than WT. Conversely, root tissues of transgenic lines displayed a ‘high Na^+^/low K^+^’ pattern, showing significantly higher Na^+^/K^+^ ratios compared to WT (Figure 7A). Additionally, gene expression analysis at the molecular level demonstrated that *PoMPK3* expression significantly enhanced the stress responsiveness of key ion transport genes in *Arabidopsis* leaf tissue. Under salt stress, these genes (*AtSOS1*, *AtNHX1*, and *AtHKT1*) showed significantly higher expression levels in transgenic lines compared to WT. Among them, *AtSOS1* (responsible for Na^+^ efflux) and *AtNHX1* (involved in vacuolar compartmentalization) were most strongly upregulated in line #2, while *AtHKT1* (involved in xylem Na^+^ unloading) exhibited significantly enhanced expression in lines #10 and #12 (Figure 7B). The coordinated upregulation of these genes provided a direct molecular explanation for the optimized ion distribution observed between roots and leaves of *PoMPK3* transgenic *Arabidopsis* lines.

### 3.5. Integrated Multi-Transcriptomic Analysis Revealed the Transcriptional Regulatory Network Downstream of PoMPK3

**Figure 8:**
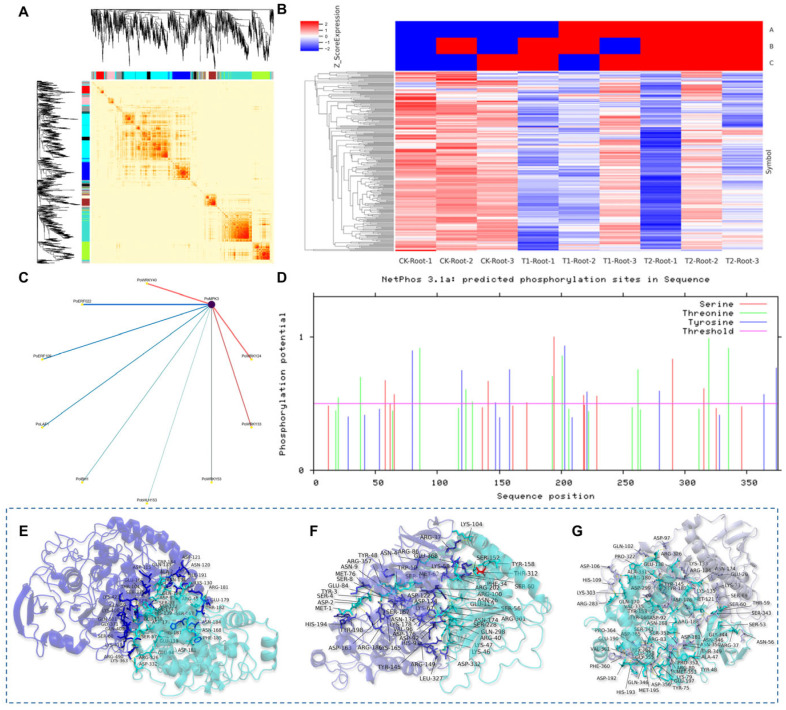
Identification of the co-expression network and molecular docking analysis of *PoMPK3* with downstream TFs in *P. oleracea* under Salt Stress. (**A**) Construction of weighted gene co-expression network and module identification. The network was constructed using a soft-thresholding power to satisfy scale-free topology. Genes were clustered into 11 distinct co-expression modules, each represented by a unique color (the grey module contained 151 genes that could not be clustered and lacked biological relevance). (**B**) Expression profiling of *PoMPK3* and its co-expressed DEGs under salt stress. A heatmap displayed the expression patterns (FPKM value) of the 167 DEGs from the green–yellow module (including *PoMPK3*) across different experimental conditions (CK, T1, T2 in roots/leaves). A stringent screening criterion was applied to identify DEGs with strong salt stress responsiveness as tightly co-expressed candidates. The heatmap columns were grouped into three clusters (labeled A, B, and C on the right), which correspond to the control (CK), early salt stress (T1), and late salt stress (T2) conditions, respectively. Data represents SD (*n* = 3 biological replicates). (**C**) Screening of transcription factors co-expressed with *PoMPK3* and their interaction network. Nine TFs were identified from the candidate DEGs. A weighted correlation network highlighted TFs as having the most significant co-expression relationships (highest weight coefficient) with *PoMPK3*. Node size reflected connectivity degree, while edge thickness indicated co-expression weight. (**D**) In silico prediction of potential phosphorylation sites on the PoMPK3 protein. The NetPhos server predicted numerous high-confidence phosphorylation sites (score > 0.7) on serine (S), threonine (T), and tyrosine (Y) residues. The predicted kinases responsible for these modifications include PKC, CKII, PKA, GSK3, CaMKII, and CDC2. Molecular docking model of the (**E**) P oMPK3–PoWRKY33, (**F**) PoMPK3–PoWRKY40, and (**G**) PoMPK3–PoWRKY53 complexes and prediction of phosphorylation sites. In these models, the PoMPK3 protein was represented as a solid surface colored in a consistent shade of cyan. The interacting partners are shown as cartoon representations in purple (E), dark blue (F), and light grey (G), respectively. Key residues at the predicted interaction interfaces were shown as sticks and labeled.

To explore the molecular regulatory network through which *PoMPK3* enhanced plant salt tolerance, we performed WGCNA using transcriptome data from leaves and roots of *P. oleracea* at different time points under salt stress (Supplementary Figure S3), aiming to identify downstream TFs potentially regulated by *PoMPK3*-mediated phosphorylation. After preprocessing the raw data by removing entries with missing gene expression values, applying quantile normalization, and performing feature selection using the WGCNA package, a refined dataset of 1443 genes was obtained for downstream analysis. A co-expression network was constructed using optimized soft-thresholding power, and genes were classified into 11 distinct modules (Supplementary Table S2, and the grey module lacked biological relevance) (Figure 8A). Search results indicated that the *PoMPK3* gene was located within the green–yellow module (Supplementary Table S3), which contained a total of 167 DEGs. These DEGs were further compared with microarray datasets under salt stress conditions. A stringent screening criterion was applied, wherein only DEGs exhibiting strong salt stress responsiveness were considered tightly co-expressed candidates (Figure 8B). Subsequently, nine TFs were identified from these candidate DEGs. Weighted correlation analysis revealed that *PoWRKY33*, *PoWRKY40*, and *PoWRKY53* exhibited the most significant co-expression relationships with *PoMPK3* (Figure 8C).

Based on NetPhos prediction, the PoMPK3 protein sequence contained numerous potential phosphorylation sites, including multiple high-confidence serine, threonine, and tyrosine residues. The predicted kinases responsible for these modifications belonged to key kinases, including PKC, CKII, PKA, GSK3, CaMKII, and CDC2 (Figure 8D). Molecular docking simulations predicted that PoMPK3 and PoWRKY33 formed a stable complex with a binding energy of −8.2 kcal mol^−1^, where ASP-121 in PoMPK3 (located in the activation loop) formed a salt bridge (2.8 Å) with LYS-39 of PoWRKY33 while positioning 3.2 Å from SER-37’s hydroxyl group in PoWRKY33, with secondary stabilization through hydrogen bonds between THR-214 (PoMPK3) and SER-37 (PoWRKY33) (3.1 Å) and hydrophobic contacts involving LEU-198 (PoMPK3) and PHE-41 (PoWRKY33) (Figure 8E). At the same time, it was predicted that PoMPK3 and PoWRKY40 formed a stable complex (−7.6 kcal mol^−1^), with THR-312 of PoWRKY40 positioned 3.1 Å from the catalytic center. Secondary stabilization involved hydrogen bonds between ASN-212 (PoMPK3) and GLN-127 (PoWRKY40), and hydrophobic contacts of TYR-198 (PoMPK3) with TYR-204 (PoWRKY40) (Figure 8F). In addition, as shown in Figure 8G, PoMPK3 and PoWRKY53 formed a stable complex with a binding energy of −7.9 kcal mol^−1^, indicating strong spontaneous interaction. The extensive interface involved key residues including SER-351 and THR-407 in PoWRKY53, positioned within 3.2/3.5 Å of the catalytic center of PoMPK3, making them high-confidence phosphorylation targets. Additional stabilization was provided by salt bridge formation between ASP-121 (PoMPK3) and LYS-410 (PoWRKY53), hydrogen bonding network involving ASN-212 (PoMPK3) and GLN-351 (PoWRKY53), and hydrophobic contacts between TYR-198 (PoMPK3) and PHE-408 (PoWRKY53).

## 4. Discussion

The classification of plant MAPKs into TEY and TDY subtypes is based on the central amino acid residue present in the TXY motif [9]. Group A MAPKs, which include MPK3 and MPK6, are known to participate in plant immunity and abiotic stress responses [47]. Our transcriptomic screening under salt stress identified seven MPK family members in *P. oleracea*, among which *PoMPK3* emerged as a core candidate due to its markedly higher and root-specific upregulation. These findings supported the important role of *PoMPK3*, a Group A MAPK, in the initial salt stress response in roots, leading to its selection for detailed study. In contrast, Group B MAPKs, such as *MPK4*, *MPK5*, *MPK11*, *MPK12*, and *MPK13*, are known to function in plant immunity adaptation and developmental regulation [48,49,50]. In addition, group C MAPKs (including MPK1, MPK2, MPK7, and MPK14) have been less thoroughly studied [51,52]. Our subcellular localization results demonstrated that PoMPK3 underwent specific membrane trafficking and contained an intrinsic membrane-targeting signal, providing crucial structural insights into its activation mechanism. This membrane association is functionally important as it positions PoMPK3 near its upstream activators, the membrane-anchored MAPKKs [53]. This spatial arrangement enables rapid and efficient phosphorylation of the conserved T-E-Y motif in the PoMPK3 activation loop during the salt stress response. The high degree of conservation observed in the T-E-Y motif underscores the functional importance of this activation loop. This evolutionary preservation across diverse species, from *Arabidopsis* [54] to major crops like rice [55] and wheat [56], strongly suggests that *PoMPK3* operates through a core signaling mechanism that is fundamental to plant salt stress responses.

Meanwhile, the phosphorylation of key TFs like *PoWRKY33*, *PoWRKY40*, and *PoWRKY53*, as predicted by our molecular docking analysis, exemplified the relay of this activated signal into the nucleus to orchestrate gene expression changes. The WRKY gene family represents a class of TFs unique to plants, known to play significant roles in regulating plant responses to saline–alkali stress [57]. The *Arabidopsis* transcription factor *AtWRKY33*, an upstream regulator of *AtCYP94B1*, enhanced salt tolerance by facilitating apoplastic barrier formation through increased root suberin deposition, whereas its silencing led to reduced suberin and a salt-sensitive phenotype [58]. In *Fortunella crassifolia*, *FcWRKY40* functioned as a positive regulator in the ABA signaling pathway, enhancing plant salt tolerance through the regulation of genes involved in ion homeostasis and proline biosynthesis [59]. Conversely, *OsWRKY53* acted as a negative regulator of salt tolerance in rice, suppressing the expression of genes critical for ion homeostasis [60]. Our findings indicated that the predicted high-confidence phosphorylation sites on these TFs (SER-37 in PoWRKY33, THR-312 in PoWRKY40, and SER-351/THR-407 in PoWRKY53) were strategically located near their DNA-binding domains and were spatially oriented toward the catalytic cleft of PoMPK3. This implies that phosphorylation by PoMPK3 was poised to directly modulate the DNA-binding affinity, transcriptional activity, or protein stability of these TFs, thereby reprogramming the stress-responsive transcriptome [61]. However, it is important to note that the physical interaction between PoMPK3 and these WRKY TFs, as well as the specific phosphorylation events, were based on computational predictions. While our docking analysis provided high-confidence predictions, experimental validation through co-immunoprecipitation (co-IP) and bimolecular fluorescence complementation (BiFC) assays are required to confirm these interactions in vivo. Particularly, the predicted phosphorylation of WRKYs at Ser/Thr residues (such as Thr-312 in PoWRKY40) needs direct evidence from pull-down assays combined with mass spectrometry.

It was observed that compared to WT, heterologous expression of *PoMPK3* in *A. thaliana* significantly upregulated the expression of key ABA pathway genes and improved the accumulation of endogenous ABA, clearly demonstrating that *PoMPK3* functioned upstream of ABA biosynthesis and signaling. This ABA response was particularly pronounced in the strongest transgenic line (line #2), establishing a positive correlation between *PoMPK3* expression level and the magnitude of ABA pathway activation. Therefore, we hypothesized that this activation was mechanistically achieved through the direct phosphorylation of downstream TFs by *PoMPK3*. Based on our molecular docking predictions, TFs such as *WRKY33* were high-confidence substrates of PoMPK3 [62]. Phosphorylated WRKY33 (or WRKY40) functioned as a transcriptional activator, binding to the promoter of the rate-limiting ABA biosynthetic gene *NCED3*, thereby driving its expression and initiating ABA biosynthesis. This model is strongly supported by the observed salt-stress-induced transcriptional upregulation of *AtNCED3* in our transgenic lines. Similar findings have also been reported in *Zea mays* [63], *Ipomoea trifida* [64], and *Solanum lycopersicum* [65]. The subsequent rise in ABA levels initiates a powerful positive feedback loop. This process involves ABA binding to receptors such as PYR1, which relieves the inhibition of SnRK2 kinases. The activated SnRK2s then phosphorylate and activate ABF transcription factors (*AtABF3*) [66]. These activated ABFs, potentially in concert with other PoMPK3-phosphorylated TFs, further amplify the expression of *NCED3* and other ABA-responsive genes, creating a robust, self-reinforcing signaling cascade that drives the stress adaptation process [67]. Furthermore, although transgenic studies demonstrated *PoMPK3*’s sufficiency for salt tolerance, the lack of loss-of-function evidence in *P. oleracea* (CRISPR knockout or RNAi silencing) limits causal inference.

ABA functions as a central signaling molecule in the plant stress response network, directly activating the transcription of key antioxidant enzyme genes and reinforcing ROS scavenging pathways [68]. In this study, this was robustly supported by our data showing that transgenic lines exhibited dramatically higher activities of key antioxidant enzymes (SOD, CAT, APX) and consequently, significantly lower levels of oxidative damage markers (MDA, H_2_O_2_, O_2_^−^) compared to WT. It has been reported that in *A. thaliana*, AtMPK3 perceived ABA and H_2_O_2_ signals in leaves, thereby regulating stomatal aperture [69]. In addition, *AtMPK9* and *AtMPK12* in *Arabidopsis* have been found to be key components in the defense cell ABA signaling pathway, responsible for converting oxidative signals into activation of anion channels, thereby driving stomatal closure [70]. Therefore, we speculate that the phosphorylation of WRKYs by *PoMPK3* may enhance their ability to bind to the promoters of genes encoding these antioxidant enzymes, thereby directly converting the elevated ABA signal into strong ROS scavenging ability. In addition, we also found that under salt stress, *PoMPK3* transgenic *Arabidopsis* lines accumulated higher levels of proline, betaine, soluble sugar, and soluble protein. According to previous studies, MPK3 can directly determine the rate of proline synthesis by phosphorylating transcription factors and regulating the promoter expression of the proline synthesis rate-limiting enzyme *P5CS* gene [71,72]. It can also affect ABA synthesis, upregulate *P5CS* expression, and inhibit the activity of proline degrading enzyme ProDH [56]. Furthermore, the simultaneous upregulation of betaine, soluble sugars, and soluble proteins in *PoMPK3* expressing lines was a metabolic hallmark of enhanced salt tolerance. These results indicated that *PoMPK3*-induced ABA accumulation confers salt tolerance by enhancing betaine production for protein and photosystem stability [73] and increasing soluble sugars and proteins for osmotic adjustment and cellular hydration [74]. Interestingly, the ion content analysis provided a clear and direct physiological explanation for the enhanced salt tolerance observed in *PoMPK3* expression *Arabidopsis* lines. The transgenic lines exhibited a distinct ion distribution pattern, characterized by a significantly lower Na^+^/K^+^ ratio in shoots and a higher ratio in roots compared to WT, indicating more effective ionic stress management. The pattern indicated a restriction of toxic Na^+^ accumulation in the photosynthetic tissues (shoots), thereby protecting the core metabolic machinery, while simultaneously suggesting a potential sequestration or retention of Na^+^ in the roots [75]. Meanwhile, the coordinated upregulation of key ion transporter genes in the transgenic lines provided a molecular basis for the observed ion distribution pattern. The significantly enhanced expression of *AtSOS1* and *AtNHX1* in line #2 was particularly noteworthy. *AtSOS1* encoded a plasma membrane Na^+^/H^+^ antiporter responsible for pumping Na^+^ out of the cell [43], while *AtNHX1* encoded a vacuolar membrane transporter that sequesters Na^+^ into the vacuole [76]. The simultaneous increase in the expression of these two genes provided a straightforward molecular explanation for the ‘low Na^+^’ phenotype in the shoots, as these activities collectively reduced the cytosolic Na^+^ concentration. In addition, the differential expression of *AtHKT1* inhibited Na^+^ uptake and its translocation to shoots, thereby reducing Na^+^ toxicity in photosynthetic tissues [77]. While our study utilized single baseline control and fixed sampling times to mitigate circadian effects [21], we acknowledge that the inclusion of time-matched control groups would provide an even higher resolution of the stress-specific transcriptomic response. In summary, we propose that under salt stress, *PoMPK3* is activated and phosphorylates downstream TFs (SER-57 of PoWRKY33, THR-312 of PoWRKY40, and SER-351/THR-407 of PoWRKY53), thereby coordinately triggering ABA signaling. This leads to a significant increase in endogenous ABA levels, particularly in transgenic line #2, and upregulates key ABA pathway genes (*AtNCED3*, *AtPYR1*, and *AtABF3*). The enhanced ABA signaling activates both enzymatic and non-enzymatic antioxidant systems. Enzymatic activities of SOD, CAT and APX are increased while compatible solutes, including proline, betaine, soluble sugars and soluble proteins, accumulate. These responses collectively reduce ROS markers (MDA, H_2_O_2_, and O_2_^−^) to levels significantly below those in WT. Furthermore, ion homeostasis is rebalanced, exhibiting a low Na^+^/high K^+^ profile in leaves and a high Na^+^/low K^+^ profile in roots. This optimized distribution is linked to coordinated upregulation of ion transporter genes, including *AtSOS1*, *AtNHX1*, and *AtHKT1*. The integration of these molecular physiological and biochemical adaptations translates to superior salt tolerance in transgenic *Arabidopsis*. This is demonstrated by improved seed germination rates, longer primary roots, greater biomass accumulation and enhanced survival capacity evidenced by reduced water loss, lower relative electrolyte leakage, and higher chlorophyll content under salt stress (Supplementary Figure S4).

## 5. Conclusions

Overall, this study identified and characterized PoMPK3, a Group A MAPK kinase from *P. oleracea*, as a central regulator conferring comprehensive salt tolerance. *PoMPK3* expression enhanced salt tolerance of transgenic *Arabidopsis* through concurrent modulation of ABA pathway, potentiation of the antioxidant system, massive accumulation of osmoprotectants, and optimization of ion homeostasis. This was evidenced by the hyper-accumulation of endogenous ABA, upregulation of *AtNCED3*/*AtPYR1*/*AtABF3*, elevated activities of SOD/CAT/APX, reduced oxidative damage (MDA/H_2_O_2_/O_2_^−^), and a favorable ion distribution pattern (low Na^+^/K^+^ ratio in shoots and high Na^+^/K^+^ ratio in roots) driven by the coordinated expression of *AtSOS1*/*AtNHX1*/*AtHKT1*. In addition, we proposed that these coordinated responses were initiated through PoMPK3-mediated phosphorylation of key WRKY transcription factors (*WRKY33*/*WRKY40*/*WRKY53*), which reprograms the stress-responsive transcriptome. This was demonstrated by the enhanced seed germination, vigorous root growth, increased biomass, decreased water loss rate and relative electrical conductivity, as well as increased total chlorophyll level. Therefore, our work elucidates the pivotal role of *PoMPK3* in salt tolerance, and future studies should focus on developing *PoMPK3*-based genetic markers for plant breeding programs.

## Data Availability

The original contributions presented in this study are included in the article/Supplementary Material. Further inquiries can be directed to the corresponding author.

## Supplementary Materials

The following supporting information can be downloaded at: https://www.mdpi.com/article/10.3390/plants14223478/s1, Figure S1: The flowchart summarizes the methodology of this study; Figure S2: Identification of conserved protein motifs in the MAPK gene family of *P. oleracea*. (A) Sequence logo of the single, significantly enriched motif (Motif 1) discovered in all PoMPK protein sequences. The logo was generated by the MEME Suite, with the height of each amino acid letter representing its relative frequency at that position. The analysis was stopped after identifying one motif as requested. The consensus amino acid sequence for Motif 1 is shown below. (B) Distribution of Motif 1 across the seven identified PoMPK proteins. The name of each gene and the corresponding exceptionally significant E-value (ranging from 10^−53 to 10^−56) for the motif match are listed. The red bar represents the position of the conserved motif within each protein sequence. This high degree of conservation suggests this motif constitutes a critical functional domain, likely the canonical protein kinase domain characteristic of MAPKs; Figure S3: Construction of a co-expression network for genes associated with salt-resistance physiological indices in *P. oleracea*. (A) Identification of gene clusters in WGCNA through average linkage hierarchical clustering, based on the topological overlap measure (TOM) dissimilarity. To elucidate the distinct co-expressed gene modules in *P. oleracea* roots under salt stress conditions, we conducted a weighted gene co-expression network analysis (WGCNA) using the core DEGs identified through Venn diagram analysis. After preprocessing the raw data by removing entries with missing gene expression values, applying quantile normalization, and performing feature selection using the WGCNA package, a refined dataset was obtained for downstream analysis. A weighted gene co-expression network was constructed using the optimized power value, resulting in the classification of genes into 11 modules, with the grey module having no reference significance due to its non-assignment to any module (B) Module eigengenes defined by the dynamic tree was identified by colors, including red, turquoise, blue, and others. The correlation between module eigengenes and antioxidant indices was analyzed, with each cell in the heatmap displaying the corresponding correlation coefficient and *P*-value. Search results indicated that the PoMPK3 gene was located within the greenyellow module, which contained a total of 167 DEGs; Figure S4: Potential regulatory mechanism of *PoMPK3* promoting plant salt tolerance. Besides, in the model, solid lines represent mechanistically established pathways supported by direct experimental evidence from this study. Dashed lines indicate predicted interactions based on high-confidence computational analyses (including gene co-expression, molecular docking) and literature support, which require future experimental validation. Table S1: Primer information for qRT-PCR determination; Table S2: Statistics of the number of genes in the module of WGCNA; Table S3: TFs and functional genes in module greenyellow; Table S4: mANOVA analysis of raw data in this study; Table S5: The detailed docking scores and parameters of predicted TFs; Table S6: The melting curve and primer amplification efficiency.

## Abbreviations

ABA	Abscisic Acid
ANOVA	Analysis of Variance
APX	Ascorbate Peroxidase
CAT	Catalase
FPKM	Fragments Per Kilobase Million
GO	Gene Ontology
H_2_O_2_	Hydrogen Peroxide
ICP-MS	Inductively Coupled Plasma- Mass Spectrometry
KEGG	Kyoto Encyclopedia of Genes and Genomes
MAPK	Mitogen-Activated Protein Kinase
MDA	Malondialdehyde
O_2_^−^	Superoxide Anion
PBS	Phosphate-Buffered Saline
REC	Relative Electrolyte Conductivity
ROS	Reactive Oxygen Species
SOD	Superoxide Dismutase
TOM	Topological Overlap Matrix
WGCNA	Weighted Gene Co-expression Network Analysis

## References

[1] Verma K.K., Song X.P., Kumari A., Jagadesh M., Singh S.K., Bhatt R., Singh M., Seth C.S., Li Y.R. (2025). Climate change adaptation: Challenges for agricultural sustainability. Plant Cell Environ..

[2] Lee D.G., Ahn K.H. (2022). Assessment of Suitable Gridded Climate Datasets for Large-Scale Hydrological Modelling over South Korea. Remote Sens..

[3] Liang X., Li J., Yang Y., Jiang C., Guo Y. (2024). Designing salt stress-resilient crops: Current progress and future challenges. J. Integr. Plant Biol..

[4] van Zelm E., Zhang Y., Testerink C. (2020). Salt Tolerance Mechanisms of Plants. Annu. Rev. Plant Biol..

[5] Chen X., Ding Y., Yang Y., Song C., Wang B., Yang S., Guo Y., Gong Z. (2021). Protein kinases in plant responses to drought, salt, and cold stress. J. Integr. Plant Biol..

[6] Zhu J.K. (2002). Salt and drought stress signal transduction in plants. Annu. Rev. Plant Biol..

[7] Anastácio A., Carvalho I.S. (2013). Accumulation of fatty acids in purslane grown in hydroponic salt stress conditions. Int. J. Food Sci. Nutr..

[8] Miao L., Tao H., Peng Y., Wang S., Zhong Z., El-Seedi H., Dragan S., Zengin G., Cheang W.S., Wang Y. (2019). The anti-inflammatory potential of *Portulaca oleracea* L. (purslane) extract by partial suppression on NF-κB and MAPK activation. Food Chem..

[9] Zhang M., Zhang S. (2022). Mitogen-activated protein kinase cascades in plant signaling. J. Integr. Plant Biol..

[10] Enders T.A., Frick E.M., Strader L.C. (2017). An Arabidopsis kinase cascade influences auxin-responsive cell expansion. Plant J..

[11] Hu L., Ye M., Li R., Zhang T., Zhou G., Wang Q., Lu J., Lou Y. (2015). The Rice Transcription Factor WRKY53 Suppresses Herbivore-Induced Defenses by Acting as a Negative Feedback Modulator of Mitogen-Activated Protein Kinase Activity. Plant Physiol..

[12] Borsai O., Hassan M.A., Negrușier C., Raigón M.D., Boscaiu M., Sestraș R.E., Vicente O. (2020). Responses to Salt Stress in *Portulaca*: Insight into Its Tolerance Mechanisms. Plants.

[13] Rodrigues Neto J.C., Salgado F.F., Braga Í.O., Carvalho da Silva T.L., Belo Silva V.N., Leão A.P., Ribeiro J.A.A., Abdelnur P.V., Valadares L.F., de Sousa C.A.F. (2023). Osmoprotectants play a major role in the *Portulaca oleracea* resistance to high levels of salinity stress-insights from a metabolomics and proteomics integrated approach. Front. Plant Sci..

[14] Farooq M.A., Zeeshan Ul Haq M., Zhang L., Wu S., Mushtaq N., Tahir H., Wang Z. (2024). Transcriptomic Insights into Salt Stress Response in Two Pepper Species: The Role of MAPK and Plant Hormone Signaling Pathways. Int. J. Mol. Sci..

[15] Su Y., Guo A., Huang Y., Wang Y., Hua J. (2020). *GhCIPK6a* increases salt tolerance in transgenic upland cotton by involving in ROS scavenging and MAPK signaling pathways. BMC Plant Biol..

[16] Fujii H., Zhu J.K. (2009). Arabidopsis mutant deficient in 3 abscisic acid-activated protein kinases reveals critical roles in growth, reproduction, and stress. Proc. Natl. Acad. Sci. USA.

[17] Aslam M., Greaves J.G., Jakada B.H., Fakher B., Wang X., Qin Y. (2022). AcCIPK5, a pineapple CBL-interacting protein kinase, confers salt, osmotic and cold stress tolerance in transgenic *Arabidopsis*. Plant Sci..

[18] Shen T., Xu F., Chen D., Yan R., Wang Q., Li K., Zhang G., Ni L., Jiang M. (2024). A B-box transcription factor OsBBX17 regulates saline-alkaline tolerance through the MAPK cascade pathway in rice. New Phytol..

[19] Xing J.C., Zhao B.Q., Dong J., Liu C., Wen Z.G., Zhu X.M., Ding H.R., He T.T., Yang H., Wang M.W. (2020). Transcriptome and Metabolome Profiles Revealed Molecular Mechanisms Underlying Tolerance of *Portulaca oleracea* to Saline Stress. Russ. J. Plant Physiol..

[20] Khan S., Rowe S.C., Harmon F.G. (2010). Coordination of the maize transcriptome by a conserved circadian clock. BMC Plant Biol..

[21] Covington M.F., Maloof J.N., Straume M., Kay S.A., Harmer S.L. (2008). Global transcriptome analysis reveals circadian regulation of key pathways in plant growth and development. Genome Biol..

[22] Zhang X., Henriques R., Lin S.S., Niu Q.W., Chua N.H. (2006). *Agrobacterium*-mediated transformation of *Arabidopsis thaliana* using the floral dip method. Nat. Protoc..

[23] Langfelder P., Horvath S. (2008). WGCNA: An R package for weighted correlation network analysis. BMC Bioinform..

[24] Xu S., Hu E., Cai Y., Xie Z., Luo X., Zhan L., Tang W., Wang Q., Liu B., Wang R. (2024). Using clusterProfiler to characterize multiomics data. Nat. Protoc..

[25] Shannon P., Markiel A., Ozier O., Baliga N.S., Wang J.T., Ramage D., Amin N., Schwikowski B., Ideker T. (2003). Cytoscape: A software environment for integrated models of biomolecular interaction networks. Genome Res..

[26] Sievers F., Wilm A., Dineen D., Gibson T.J., Karplus K., Li W., Lopez R., McWilliam H., Remmert M., Söding J. (2011). Fast, scalable generation of high-quality protein multiple sequence alignments using Clustal Omega. Mol. Syst. Biol..

[27] Tamura K., Stecher G., Kumar S. (2021). MEGA11: Molecular Evolutionary Genetics Analysis Version 11. Mol. Biol. Evol..

[28] Abramson J., Adler J., Dunger J., Evans R., Green T., Pritzel A., Ronneberger O., Willmore L., Ballard A.J., Bambrick J. (2024). Accurate structure prediction of biomolecular interactions with AlphaFold 3. Nature.

[29] Li Y., Zhou J., Li Z., Qiao J., Quan R., Wang J., Huang R., Qin H. (2022). SALT AND ABA RESPONSE ERF1 improves seed germination and salt tolerance by repressing ABA signaling in rice. Plant Physiol..

[30] Yang T., Gan L., Peng X., Peng X., Li L., Huang Y., Xiong F., Wei M. (2025). Overexpression of cassava *MeAMY1* and *MeBAM3* genes enhance drought and salt stress tolerance in transgenic *Arabidopsis*. Plant Physiol. Biochem..

[31] Jiang W., Wang Z., Li Y., Liu X., Ren Y., Li C., Luo S., Singh R.M., Li Y., Kim C. (2024). FERONIA regulates salt tolerance in *Arabidopsis* by controlling photorespiratory flux. Plant Cell.

[32] Zhao W., Jung S., Schubert S. (2019). Transcription profile analysis identifies marker genes to distinguish salt shock and salt stress after stepwise acclimation in *Arabidopsis thaliana* and *Zea mays*. Plant Physiol. Biochem..

[33] Sun M., Qiao H.X., Yang T., Zhao P., Zhao J.H., Luo J.M., Liu F.F., Xiong A.S. (2024). *DcMYB62*, a transcription factor from carrot, enhanced cadmium tolerance of *Arabidopsis* by inducing the accumulation of carotenoids and hydrogen sulfide. Plant Physiol. Biochem..

[34] Guo Z., Zuo Y., Wang S., Zhang X., Wang Z., Liu Y., Shen Y. (2024). Early signaling enhance heat tolerance in *Arabidopsis* through modulating jasmonic acid synthesis mediated by HSFA2. Int. J. Biol. Macromol..

[35] Bai Y.L., Cai B.D., Luo X.T., Ye T.T., Feng Y.Q. (2018). Simultaneous Determination of Abscisic Acid and Its Catabolites by Hydrophilic Solid-Phase Extraction Combined with Ultra High Performance Liquid Chromatography-Tandem Mass Spectrometry. J. Agric. Food Chem..

[36] Li Y., Liang G., Nai G., Lu S., Ma W., Ma Z., Mao J., Chen B. (2023). VaSUS2 confers cold tolerance in transgenic tomato and *Arabidopsis* by regulation of sucrose metabolism and ROS homeostasis. Plant Cell Rep..

[37] Zhang H., Zhang K., Liu T., Zhang Y., Tang Z., Dong J., Wang F. (2022). The characterization and expression analysis under stress conditions of PCST1 in *Arabidopsis*. Plant Signal. Behav..

[38] Sun M., Qiao H.X., Yang T., Zhao P., Zhao J.H., Luo J.M., Luan H.Y., Li X., Wu S.C., Xiong A.S. (2024). Hydrogen sulfide alleviates cadmium stress in germinating carrot seeds by promoting the accumulation of proline. J. Plant Physiol..

[39] Abdelaziz M.E., Kim D., Ali S., Fedoroff N.V., Al-Babili S. (2017). The endophytic fungus *Piriformospora indica* enhances *Arabidopsis thaliana* growth and modulates Na^+^/K^+^ homeostasis under salt stress conditions. Plant Sci..

[40] Tan B.C., Joseph L.M., Deng W.T., Liu L., Li Q.B., Cline K., McCarty D.R. (2003). Molecular characterization of the *Arabidopsis* 9-cis epoxycarotenoid dioxygenase gene family. Plant J..

[41] Park S.Y., Fung P., Nishimura N., Jensen D.R., Fujii H., Zhao Y., Lumba S., Santiago J., Rodrigues A., Chow T.F. (2009). Abscisic acid inhibits type 2C protein phosphatases via the PYR/PYL family of START proteins. Science.

[42] Wang Z., Su G., Li M., Ke Q., Kim S.Y., Li H., Huang J., Xu B., Deng X.P., Kwak S.S. (2016). Overexpressing Arabidopsis ABF3 increases tolerance to multiple abiotic stresses and reduces leaf size in alfalfa. Plant Physiol. Biochem..

[43] Oh D.H., Lee S.Y., Bressan R.A., Yun D.J., Bohnert H.J. (2010). Intracellular consequences of SOS1 deficiency during salt stress. J. Exp. Bot..

[44] Chu M., Chen P., Meng S., Xu P., Lan W. (2021). The *Arabidopsis* phosphatase PP2C49 negatively regulates salt tolerance through inhibition of AtHKT1;1. J. Integr. Plant Biol..

[45] Leidi E.O., Barragán V., Rubio L., El-Hamdaoui A., Ruiz M.T., Cubero B., Fernández J.A., Bressan R.A., Hasegawa P.M., Quintero F.J. (2010). The AtNHX1 exchanger mediates potassium compartmentation in vacuoles of transgenic tomato. Plant J..

[46] Czechowski T., Stitt M., Altmann T., Udvardi M.K., Scheible W.R. (2005). Genome-wide identification and testing of superior reference genes for transcript normalization in Arabidopsis. Plant Physiol..

[47] Wu M., Wang S., Ma P., Li B., Hu H., Wang Z., Qiu Q., Qiao Y., Niu D., Lukowitz W. (2024). Dual roles of the MPK3 and MPK6 mitogen-activated protein kinases in regulating *Arabidopsis* stomatal development. Plant Cell.

[48] Verma N., Singh D., Mittal L., Banerjee G., Noryang S., Sinha A.K. (2024). MPK4-mediated phosphorylation of PHYTOCHROME INTERACTING FACTOR4 controls thermosensing by regulating histone variant H2A.Z deposition. Plant Cell.

[49] Wei J., Cui J., Zheng G., Dong X., Wu Z., Fang Y., Sa E., Zhu S., Li B., Wei H. (2025). BnaHSFA2, a heat shock transcription factor interacting with HSP70 and MPK11, enhances freezing tolerance in transgenic rapeseed. Plant Physiol. Biochem..

[50] Yeh C.Y., Wang Y.S., Takahashi Y., Kuusk K., Paul K., Arjus T., Yadlos O., Schroeder J.I., Ilves I., Garcia-Sosa A.T. (2023). MPK12 in stomatal CO_2_ signaling: Function beyond its kinase activity. New Phytol..

[51] Ren N., Zhang G., Yang X., Chen J., Ni L., Jiang M. (2024). MAPKKK28 functions upstream of the MKK1-MPK1 cascade to regulate abscisic acid responses in rice. Plant Cell Environ..

[52] Li Y., Hui S., Yuan Y., Ye Y., Ma X., Zhang X., Zhang S., Zhang C., Chen Y. (2023). PhyB-dependent phosphorylation of mitogen-activated protein kinase cascade MKK2-MPK2 positively regulates red light-induced stomatal opening. Plant Cell Environ..

[53] Shao Y., Yu X., Xu X., Li Y., Yuan W., Xu Y., Mao C., Zhang S., Xu J. (2020). The YDA-MKK4/MKK5-MPK3/MPK6 Cascade Functions Downstream of the RGF1-RGI Ligand-Receptor Pair in Regulating Mitotic Activity in Root Apical Meristem. Mol. Plant.

[54] Li H., Ding Y., Shi Y., Zhang X., Zhang S., Gong Z., Yang S. (2017). MPK3- and MPK6-Mediated ICE1 Phosphorylation Negatively Regulates ICE1 Stability and Freezing Tolerance in *Arabidopsis*. Dev. Cell.

[55] Banerjee G., Jonwal S., Rengasamy B., Pal U., Singh D., Mohit M., Sinha A.K. (2025). KRP3 Stability Controls Rice Plant Architecture and Productivity via MPK3-Mediated Phosphorylation. Plant Biotechnol. J..

[56] Liu Y., Yu T.F., Li Y.T., Zheng L., Lu Z.W., Zhou Y.B., Chen J., Chen M., Zhang J.P., Sun G.Z. (2022). Mitogen-activated protein kinase TaMPK3 suppresses ABA response by destabilising TaPYL4 receptor in wheat. New Phytol..

[57] Rai G.K., Mishra S., Chouhan R., Mushtaq M., Chowdhary A.A., Rai P.K., Kumar R.R., Kumar P., Perez-Alfocea F., Colla G. (2023). Plant salinity stress, sensing, and its mitigation through WRKY. Front. Plant Sci..

[58] Krishnamurthy P., Vishal B., Ho W.J., Lok F.C.J., Lee F.S.M., Kumar P.P. (2020). Regulation of a Cytochrome P450 Gene *CYP94B1* by WRKY33 Transcription Factor Controls Apoplastic Barrier Formation in Roots to Confer Salt Tolerance. Plant Physiol..

[59] Dai W., Wang M., Gong X., Liu J.H. (2018). The transcription factor FcWRKY40 of *Fortunella crassifolia* functions positively in salt tolerance through modulation of ion homeostasis and proline biosynthesis by directly regulating SOS2 and P5CS1 homologs. New Phytol..

[60] Zheng Y., Ge J., Bao C., Chang W., Liu J., Shao J., Liu X., Su L., Pan L., Zhou D.X. (2020). Histone Deacetylase HDA9 and WRKY53 Transcription Factor Are Mutual Antagonists in Regulation of Plant Stress Response. Mol. Plant.

[61] Schmidt R., Mieulet D., Hubberten H.M., Obata T., Hoefgen R., Fernie A.R., Fisahn J., San Segundo B., Guiderdoni E., Schippers J.H. (2013). Salt-responsive ERF1 regulates reactive oxygen species-dependent signaling during the initial response to salt stress in rice. Plant Cell.

[62] Wang S., Han S., Zhou X., Zhao C., Guo L., Zhang J., Liu F., Huo Q., Zhao W., Guo Z. (2023). Phosphorylation and ubiquitination of OsWRKY31 are integral to OsMKK10-2-mediated defense responses in rice. Plant Cell.

[63] Zhao L., Yan J., Xiang Y., Sun Y., Zhang A. (2021). ZmWRKY104 Transcription Factor Phosphorylated by ZmMPK6 Functioning in ABA-Induced Antioxidant Defense and Enhance Drought Tolerance in Maize. Biology.

[64] Sun S., Li X., Gao S., Nie N., Zhang H., Yang Y., He S., Liu Q., Zhai H. (2022). A Novel WRKY Transcription Factor from *Ipomoea trifida*, ItfWRKY70, Confers Drought Tolerance in Sweet Potato. Int. J. Mol. Sci..

[65] Chen H., Shi Y., An L., Yang X., Liu J., Dai Z., Zhang Y., Li T., Ahammed G.J. (2024). Overexpression of SlWRKY6 enhances drought tolerance by strengthening antioxidant defense and stomatal closure via ABA signaling in *Solanum lycopersicum* L. Plant Physiol. Biochem..

[66] Song J., Sun P., Kong W., Xie Z., Li C., Liu J.H. (2023). SnRK2.4-mediated phosphorylation of ABF2 regulates ARGININE DECARBOXYLASE expression and putrescine accumulation under drought stress. New Phytol..

[67] Bhagat P.K., Verma N., Pandey S., Verma D., Sinha A.K. (2025). MPK3 mediated phosphorylation inhibits the dimerization of ABI5 to fine-tune the ABA signaling in *Arabidopsis*. Plant Physiol. Biochem..

[68] Cutler S.R., Rodriguez P.L., Finkelstein R.R., Abrams S.R. (2010). Abscisic acid: Emergence of a core signaling network. Annu. Rev. Plant Biol..

[69] Gudesblat G.E., Iusem N.D., Morris P.C. (2007). Guard cell-specific inhibition of *Arabidopsis* MPK3 expression causes abnormal stomatal responses to abscisic acid and hydrogen peroxide. New Phytol..

[70] Jammes F., Song C., Shin D., Munemasa S., Takeda K., Gu D., Cho D., Lee S., Giordo R., Sritubtim S. (2009). MAP kinases MPK9 and MPK12 are preferentially expressed in guard cells and positively regulate ROS-mediated ABA signaling. Proc. Natl. Acad. Sci. USA.

[71] Verma D., Jalmi S.K., Bhagat P.K., Verma N., Sinha A.K. (2020). A bHLH transcription factor, MYC2, imparts salt intolerance by regulating proline biosynthesis in Arabidopsis. FEBS J..

[72] Mishra N.S., Tuteja R., Tuteja N. (2006). Signaling through MAP kinase networks in plants. Arch. Biochem. Biophys..

[73] Zhou Y., Guo L., Chen Z., Wang P., Zhang X., Zhao L. (2025). Enhancement of cold tolerance in tea plants (*Camellia sinensis*) by glycine betaine accumulation through CsBADH overexpression. Plant Physiol. Biochem..

[74] Kong X., Pan J., Zhang M., Xing X., Zhou Y., Liu Y., Li D., Li D. (2011). ZmMKK4, a novel group C mitogen-activated protein kinase kinase in maize (*Zea mays*), confers salt and cold tolerance in transgenic Arabidopsis. Plant Cell Environ..

[75] Choi W.G., Toyota M., Kim S.H., Hilleary R., Gilroy S. (2014). Salt stress-induced Ca^2+^ waves are associated with rapid, long-distance root-to-shoot signaling in plants. Proc. Natl. Acad. Sci. USA.

[76] Liu H.S., Liu Q., Hepworth S.R., Li P.Q., Huang J., Zhang R.X., Ma C.M., Gao T.G., Ma H.P., Ke J. (2025). ZxNHX1 from a xerophyte outperforms AtNHX1 in sequestering Na^+^ into vacuoles to enhance plant stress resistance and yield. Plant Biotechnol. J..

[77] Wang J., Luo Y., Ye F., Ding Z.J., Zheng S.J., Qiao S., Wang Y., Guo J., Yang W., Su N. (2024). Structures and ion transport mechanisms of plant high-affinity potassium transporters. Mol. Plant.

