# Supplementary material for: PoMPK3, an MAPK Gene from Purslane (Portulaca oleracea), Conferred Salt Tolerance in Transgenic Arabidopsis thaliana
Source: Plants (Basel). 2025 Nov 14;14(22):3478. doi: 10.3390/plants14223478 (PMC12656311; doi:10.3390/plants14223478)
Supplement: Supplementary file 1 [file plants-14-03478-s001.zip › plants-3950757-supplementary/revised figures and tables/Supplementary Figures.pdf]

Supplementary Figure S1. The flowchart summarizes the methodology of this study.

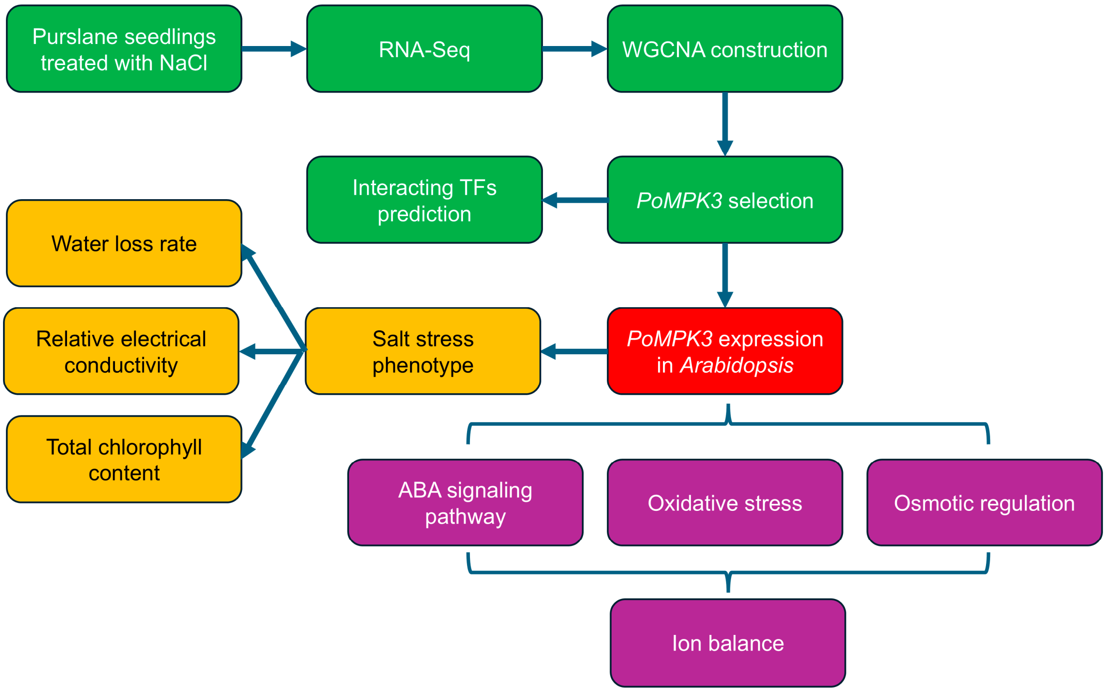

Supplementary Figure S2. Identification of conserved protein motifs in the MAPK gene family of *P. oleracea*. (**A**) Sequence logo of the single, significantly enriched motif (Motif 1) discovered in all PoMPK protein sequences. The logo was generated by the MEME Suite, with the height of each amino acid letter representing its relative frequency at that position. The analysis was stopped after identifying one motif as requested. The consensus amino acid sequence for Motif 1 is shown below. (**B**) Distribution of Motif 1 across the seven identified PoMPK proteins. The name of each gene and the corresponding exceptionally significant E-value (ranging from 10^-53 to 10^-56) for the motif match are listed. The red bar represents the position of the conserved motif within each protein sequence. This high degree of conservation suggests this motif constitutes a critical functional domain, likely the canonical protein kinase domain characteristic of MAPKs.

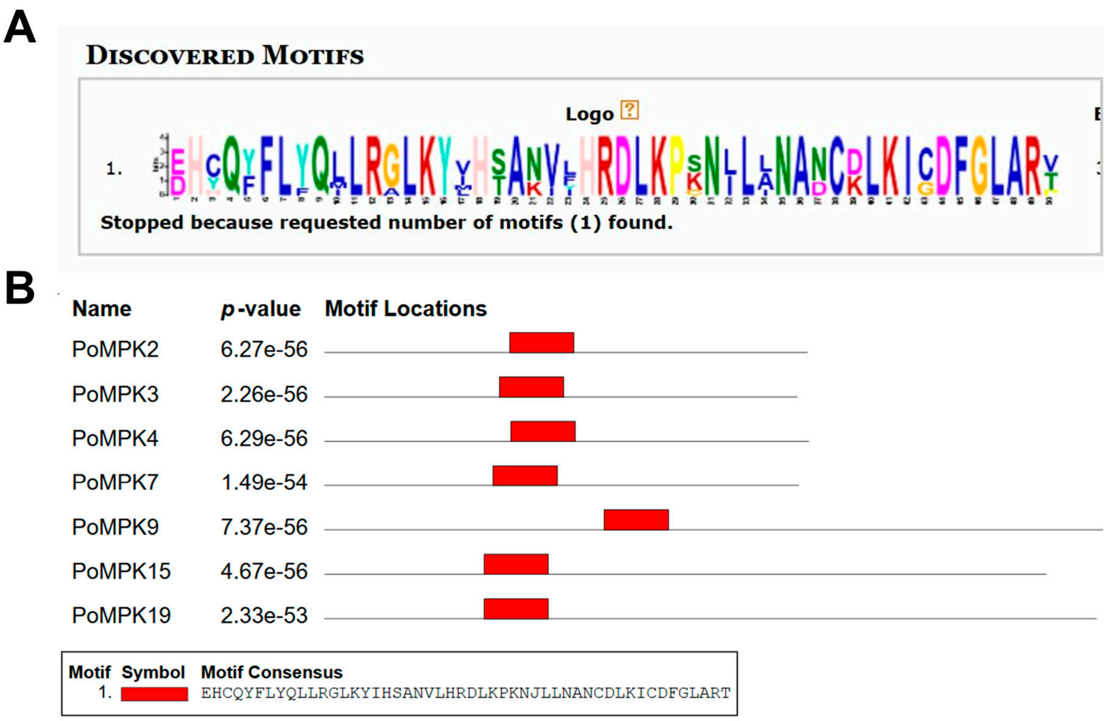

Supplementary Figure S3. Construction of a co-expression network for genes associated with salt-resistance physiological indices in *P. oleracea*. (A) Identification of gene clusters in WGCNA through average linkage hierarchical clustering, based on the topological overlap measure (TOM) dissimilarity. To elucidate the distinct co-expressed gene modules in *P. oleracea* roots under salt stress conditions, we conducted a weighted gene co-expression network analysis (WGCNA) using the core DEGs identified through Venn diagram analysis. After preprocessing the raw data by removing entries with missing gene expression values, applying quantile normalization, and performing feature selection using the WGCNA package, a refined dataset was obtained for downstream analysis. A weighted gene co-expression network was constructed using the optimized power value, resulting in the classification of genes into 11 modules, with the grey module having no reference significance due to its non-assignment to any module **(B)** Module eigengenes defined by the dynamic tree was identified by colors, including red, turquoise, blue, and others. The correlation between module eigengenes and antioxidant indices was analyzed, with each cell in the heatmap displaying the corresponding correlation coefficient and *P*-value. Search results indicated that the *PoMPK3* gene was located within the greenyellow module, which contained a total of 167 DEGs.

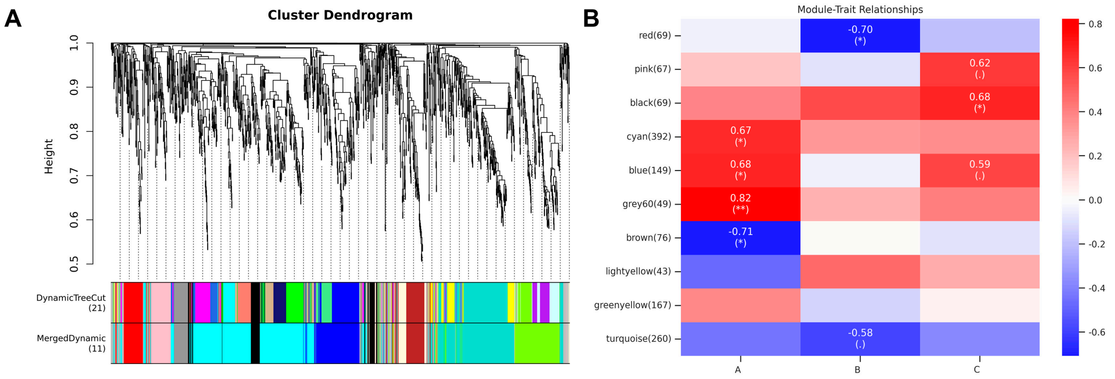

Supplementary Figure S4. Potential regulatory mechanism of *PoMPK3* promoting plant salt tolerance. Besides, in the model, solid lines represent mechanistically established pathways supported by direct experimental evidence from this study. Dashed lines indicate predicted interactions based on high-confidence computational analyses (including gene co-expression, molecular docking) and literature support, which require future experimental validation.

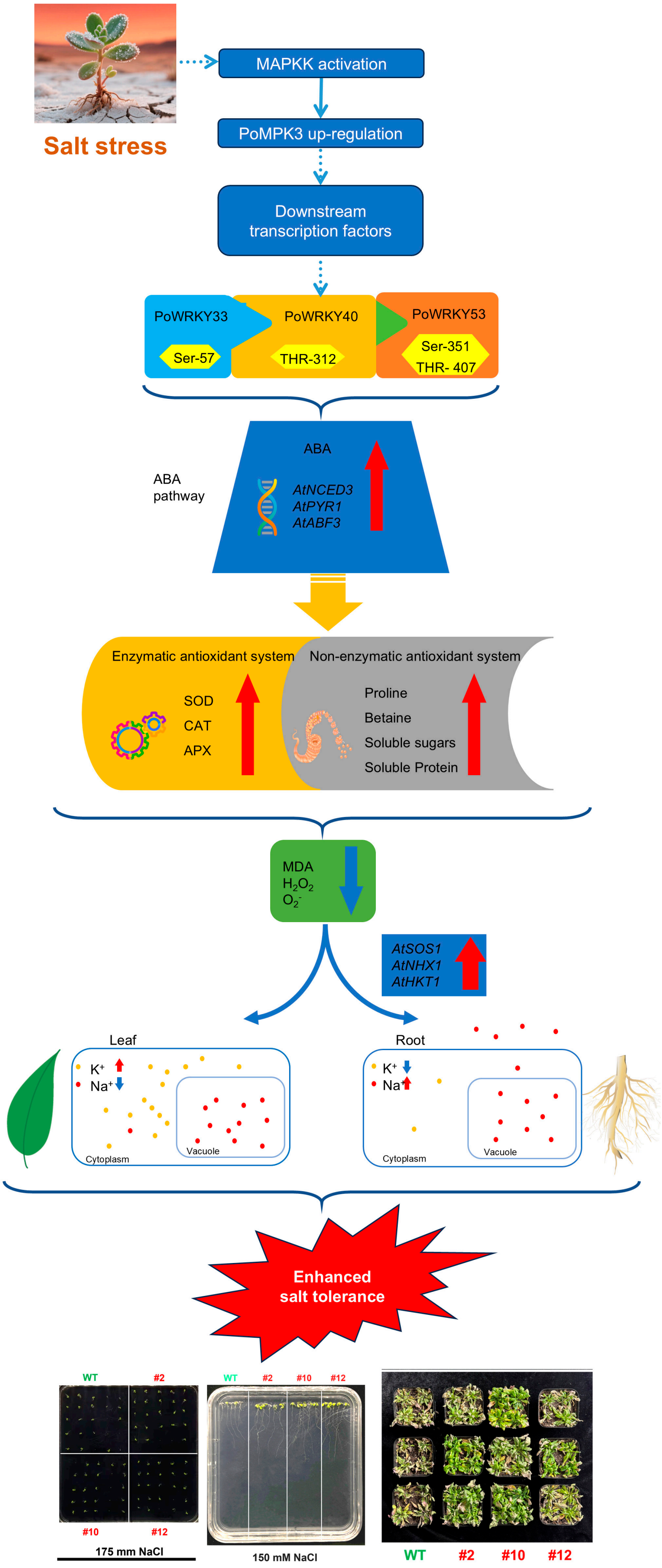

Salt stress
MAPKK activation
PoMPK3 up-regulation
Downstream transcription factors
PoWRKY33
Ser-57
PoWRKY40
THR-312
PoWRKY53
Ser-351
THR- 407
ABA pathway
ABA
AtNCED3
AtPYR1
AtABF3
Enzymatic antioxidant system
SOD
CAT
APX
Non-enzymatic antioxidant system
Proline
Betaine
Soluble sugars
Soluble Protein
MDA
H_2O_2
O_2^-
AtSOS1
AtNHX1
AtHKT1
Leaf
K^+
Na^+
Cytoplasm
Vacuole
Root
K^+
Na^+
Cytoplasm
Vacuole
Enhanced salt tolerance
WT
#2
#10
#12
175 mm NaCl
WT
#2
#10
#12
150 mM NaCl
WT
#2
#10
#12
